# Supporting employers and their employees with Mental hEalth problems to remain eNgaged and producTive at wORk (MENTOR): A feasibility randomised controlled trial protocol

**DOI:** 10.1371/journal.pone.0283598

**Published:** 2023-04-20

**Authors:** Arianna Prudenzi, Feroz Jadhakhan, Kiranpreet Gill, Michael MacArthur, Krishane Patel, Talar Moukhtarian, Charlotte Kershaw, Errin Norton-Brown, Naomi Johnston, Guy Daly, Sean Russell, Louise Thomson, Fehmidah Munir, Holly Blake, Caroline Meyer, Steven Marwaha

**Affiliations:** 1 Institute for Mental Health, School of Psychology, College of Life and Environmental Sciences, University of Birmingham, Birmingham, West Midlands, United Kingdom; 2 Warwick Manufacturing Group (WMG), School of Engineering, University of Warwick, Coventry, West Midlands, United Kingdom; 3 Warwick Medical School, Mental Health and Wellbeing Unit, University of Warwick, Coventry, West Midlands, United Kingdom; 4 Community Programmes Team, Mind, London, United Kingdom; 5 Faculty of Health and Life Sciences, Coventry University, Coventry, West Midlands, United Kingdom; 6 West Midlands Combined Authority, Birmingham, West Midlands, United Kingdom; 7 Centre for Organisational Health and Development, School of Medicine, University of Nottingham, Nottingham, West Midlands, United Kingdom; 8 School of Sport, Exercise and Health Sciences, Loughborough University, Loughborough, West Midlands, United Kingdom; 9 School of Health Sciences, University of Nottingham, Nottingham, United Kingdom; 10 NIHR Nottingham Biomedical Research Centre, Nottingham, United Kingdom; 11 Specialist Mood Disorders Clinic, The Zinnia Centre, Birmingham and Solihull Mental Health NHS Foundation Trust, Birmingham, West Midlands, United Kingdom; UNITED KINGDOM

## Abstract

Employees with mental health problems often struggle to remain in employment. During the COVID-19 pandemic, these employees face multiple additional stressors, which are likely to worsen their mental health and work productivity. Currently, it is unclear how to best support employees with mental health problems (and their managers) to improve wellbeing and productivity. We aim to develop a new intervention (MENTOR) that will jointly involve employees, managers, and a new professional (mental health employment liaison worker, MHELW), to help employees who are still at work with a mental health condition and currently receiving professional support for their mental health. A feasibility pilot study will then be undertaken to examine the feasibility and acceptability of the intervention from the perspective of employees and line managers. The study involves a feasibility randomised controlled study comparing outcomes of participants randomised to receive the intervention (MENTOR) with wait-list controls. Participants allocated to the waitlist control group will receive the intervention after three months. We aim to randomise 56 employee-manager pairs recruited from multiple organisations in the Midlands region of England. An intervention including 10 sessions for employees and managers (3 individual sessions and 4 joint sessions) will be delivered over 12 weeks by trained MHELWs. Primary outcomes include measures of feasibility and acceptability of the intervention and work productivity. Secondary outcomes include mental health outcomes. Qualitative interviews will be undertaken with a purposively selected sub-sample of employees and line managers at three-month post-intervention assessment. To our knowledge, this will be the first trial with a joint employee-manager intervention delivered by MHELWs. Anticipated challenges are dual-level consent (employees and managers), participants’ attrition, and recruitment strategies. If the intervention and trial processes are shown to be feasible and acceptable, the outcomes from this study will inform future randomised controlled trials. **Trial registration:** This trial is pre-registered with the ISRCTN registry, registration number: ISRCTN79256498. Protocol version: 3.0_March_2023. https://www.isrctn.com/ISRCTN79256498.

## Introduction

People with mental health problems find it hard to find jobs and remain in work [1–3]. Approximately one-sixth of workers experience a mental health problem at any one time [4], and anxiety and depression are considered to be responsible for almost half of working days lost in Britain due to health problems [5]. Whilst the total cost to businesses due mental health problems at work is close to £45 billion in the (United Kingdom) UK, the bulk of this is due to presenteeism (attending work whilst ill) at approximately £30 billion [6], more than three times the cost of employees’ absence. Also, evidence suggests that 12.7% of all sickness absence days in the United Kingdom (UK) can be attributed to mental health conditions which not only adds to the total cost to businesses, but has a profound impact on other areas of life, resulting in poor interpersonal relationships, difficulties in home life, low social support and high job insecurity [7–10].

Whilst a model such as international Individual Placement and Support (IPS) or the United Kingdom (UK) Access to Work programme [11] are effective at helping people to obtain work or return to work, they tend to be less successful at helping people stay in work. Also, such programmes are largely focussed on people with severe mental health problems such as schizophrenia [12,13]. Furthermore, since late 2019, the impact of the coronavirus (COVID-19) pandemic on mental health has become a serious concern. It is predicted that approximately 41.8% of the UK population is at high risk of mental health problems because of the economic vulnerability as a direct result of the COVID-19 outbreak [14]. It is highly likely that over the next few years, the long-term implications of COVID-19 and its effects on the psychological wellbeing of those who lived through the pandemic will come to light. For example, burnout, traumatic stress, anxiety, and depressive symptoms were reported after the SARS outbreak, which had similar presentations and novelty to COVID-19, suggesting that pandemics have long-term implications for mental health [15].

People who have mental health problems and are receiving treatment from health or social care services often do not receive the support at work to remain engaged and productive in their employed role [16]. This may be particularly the case in Small and Medium-sized Enterprises (SMEs) (with 10–250 employees), where employees may not be able to access Occupational Health assistance readily [16]. In the UK, SMEs (small and medium-sized enterprises) account for 99.9% of the business population (5.9 million businesses) (figures from gov.uk). Employees may not always feel comfortable disclosing unmanageable stress or poor mental health to their employers or managers [17]. How to help individuals with mental health problems remain at work and improve their, and thereby their business’ productivity is unclear, and there are major knowledge gaps in this area. In order to address this, we aim to: a] develop a new complex intervention (MENTOR) to assist employees and line managers to support employees’ wellbeing and remain productive at work; b] undertake a feasibility pilot study to examine the feasibility and acceptability of MENTOR to employees and line managers and provide preliminary estimates of the effectiveness of the intervention on productivity and mental health symptoms.

## Method

### Study design

**Fig 1:**
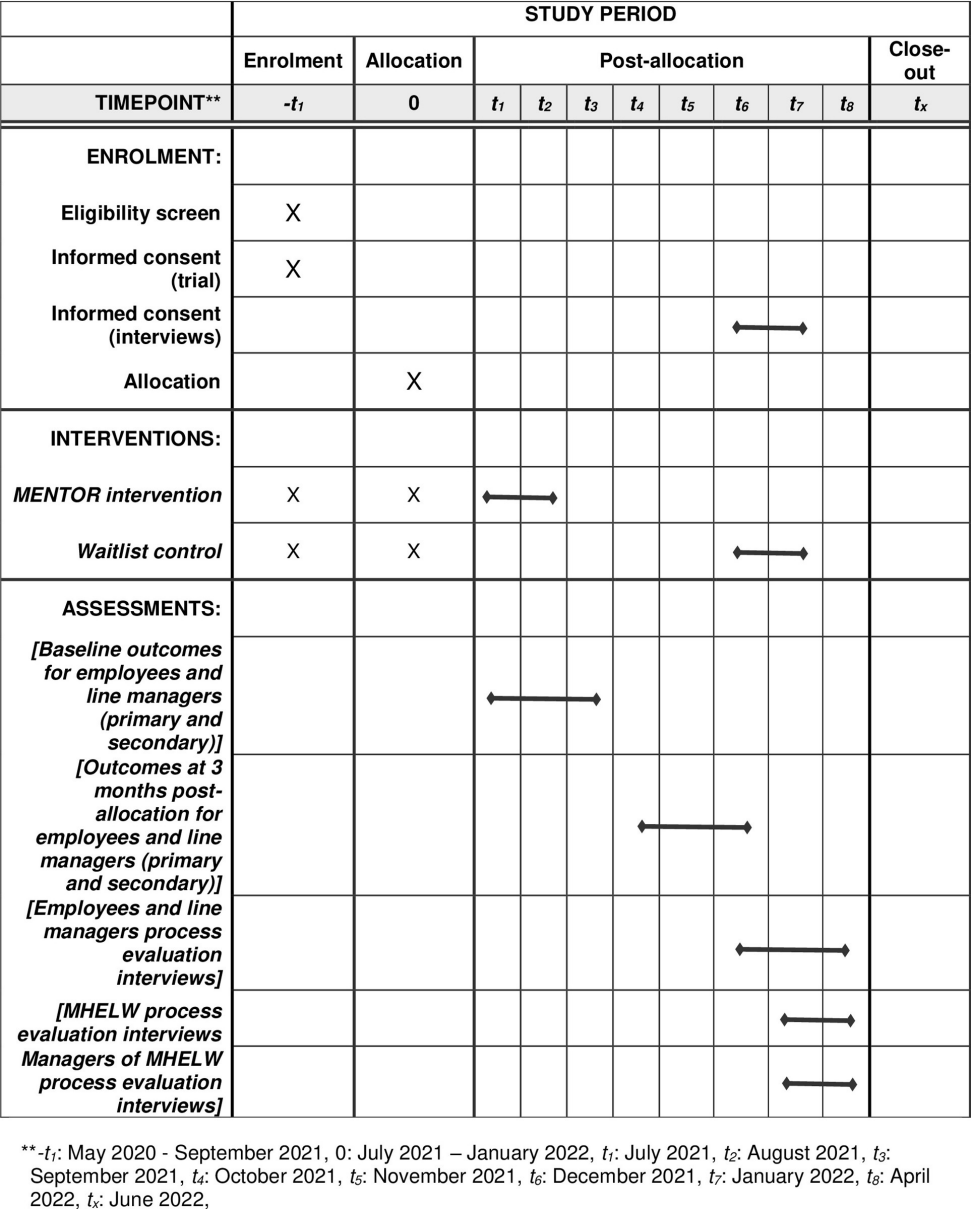
Recommended content for the schedule of enrolment, interventions, and assessments.

A feasibility randomised controlled trial with a 3-month intervention (called MENTOR) aimed at supporting workers with a clinical diagnosis of a mental disorder currently receiving healthcare treatment to remain engaged and productive at work. The intervention will be compared to a waitlist control group (a type of control group where participants randomised to the control group start their intervention once participants allocated to the intervention group complete theirs). The control arm will receive the same intervention after a 3-month delay (Fig 1). During the waiting time, participants will be allowed to seek care in both emergency and non-emergent scenarios.

**Fig 2:**
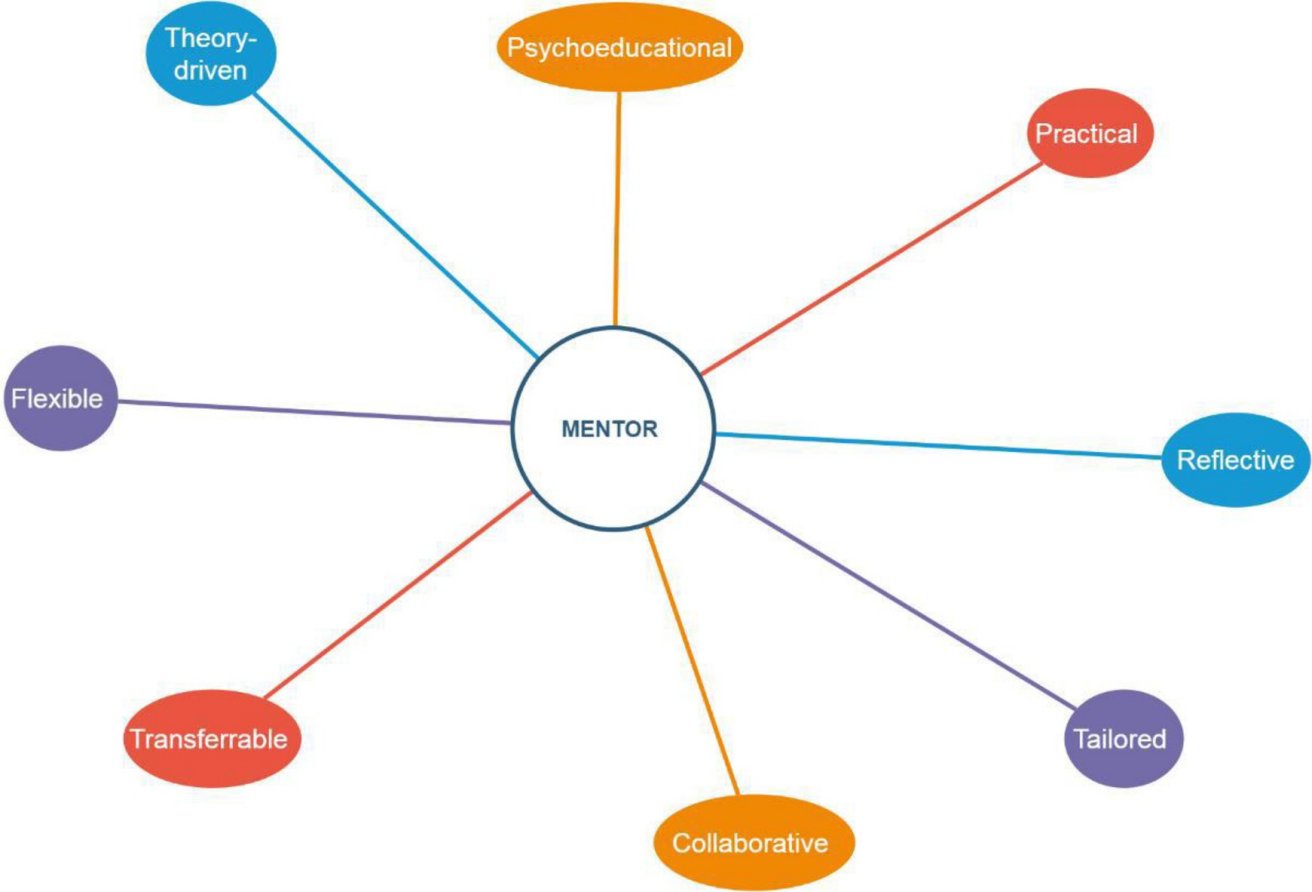
Principles of MENTOR.

The trial was pre-registered in the ISRCTN registry: https://www.isrctn.com/ISRCTN79256498 (Fig 2).

### Population

Adult workers self-identified with a clinical diagnosis of a mental health disorder, who are currently receiving treatment by a UK National Health Service (NHS) healthcare provider. Participants will be asked to state their mental health condition.

#### Inclusion criteria

▪ Full-time or part-time employees who self-identify with a clinical diagnosis of a mental health disorder (e.g., depression, bipolar disorder, substance abuse, eating disorder)
▪ Currently receiving treatment for a mental health disorder through UK NHS services
▪ Aged ≥18 years including workers that are past retirement age (the age at which participants can retire and can be taken earlier or later than the state pension age under the UK Pensions Act 2011)
▪ Able to give written informed consent
▪ Fluent in English

#### Exclusion criteria

▪ Currently in acute mental health crisis as defined by their clinical team
▪ Currently on extended sick leave (i.e., >4 weeks)
▪ Receiving input or engaged in another programme focussed on assisting workers with a mental health disorder (e.g., individual placement and support)
▪ Planning to retire within the next 10 months and unable to complete the intervention and the evaluation

#### Inclusion criteria for managers

- Being in a managerial relationship with the employee such as being direct line manager or senior manager or Chief Executive Officer (CEO) of the participant.

#### Exclusion criteria for managers

- Not being in a managerial relationship with the employee

### MENTOR intervention

#### Development

The intervention was developed by the University of Birmingham research team (AP lead) together with the Mental Health Charity Mind. The intervention aimed to support employers and their employees with Mental hEalth problems to remain eNgaged and producTive at wORk (MENTOR). We use this acronym here on in.

MENTOR was developed in accordance with the Medical Research Council (MRC) guidelines for developing and evaluating complex interventions [18] and workplace interventions [19] and with a theory of change development with a service design approach [20].

Three co-production workshops were held in the Midlands (England) with employees with current or recent experience of mental health care services, line managers and service or operation managers from local independent charities local Minds (franchised model of the national Mind charity working at a geographical level) running in partnership with national Mind, a leading mental health charity in England and Wales [21]. The co-production workshops explored: 1) the need for, and requirements of a new intervention such as MENTOR, 2) what participating in MENTOR would be like for each stakeholder and surface any concerns and suggestions, 3) the feedback on the proposed structure of the intervention to help shape and refine the offer, 4) understanding of the operational challenges to make running the service a success. The results of the co-production workshops indicated that there is a clear need for MENTOR. Additionally, six interviews were also conducted with healthcare practitioners to shape whether the intervention was feasible. People with lived experiences sent feedback on resources and content as well as more testing on the structure itself. A role play testing the intervention structure and content with an employee and manager was conducted by one of the researchers (AP). The scope of the role play was to understand the accessibility of the intervention content for employees and managers.

These activities altogether tested, developed and confirmed the research team’s aims that MENTOR should: 1) provide support to employees experiencing a mental health disorder, *and* their managers, to enable employees to feel well and engaged at work; 2) provide the understanding and increased awareness around mental health issues at work; 3) provide support in having open communication between managers and employees about mental health; 4) improve managers’ and employees’ resilience skills and ability to deal with challenging situations at work.

#### Theoretical model of MENTOR

**Fig 3:**
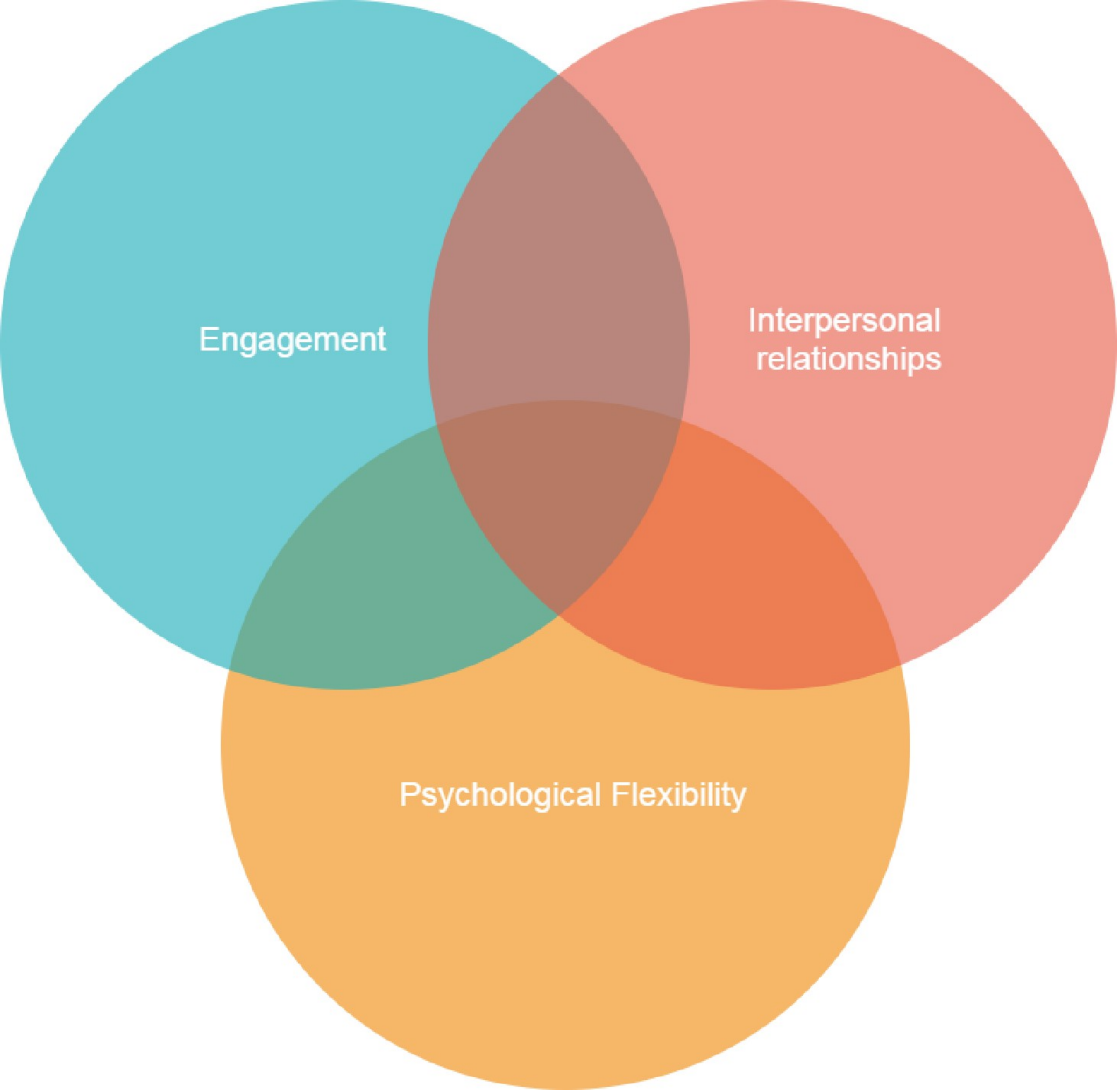
MENTOR intervention skills.

MENTOR is based on well-established theories of stress, wellbeing and burnout in the workplace, such as the Self-determination Theory [22], the Conservation of Resources Theory [23], and Contextual Behaviour Science [24]. This intervention is particularly concerned with individuals who have a mental health difficulty (e. g., have fewer personal resources to deal effectively with issues that arise within their daily life) and thus are more vulnerable at work. Therefore, the intervention aims for employees and managers to cultivate a number of resources or protective factors of health that may reduce the risk for individuals to face more serious and long-term problems (e.g., absenteeism or losing employment). The skills promoted in the intervention are: 1) Learning ways to stay well and be more productive at work (engagement), 2) Learning how to have open conversations about mental health at work (interpersonal relationships), 3) Dealing with challenging situations at work (psychological flexibility). Previous work of our team has shown that psychological flexibility is a useful psychological skill that may help people improve their mental health at work [25–29]. The intervention was manualised to facilitate training, delivery, testing and reproducibility (Figs 2 and 3).

#### Intervention format

**Fig 4:**
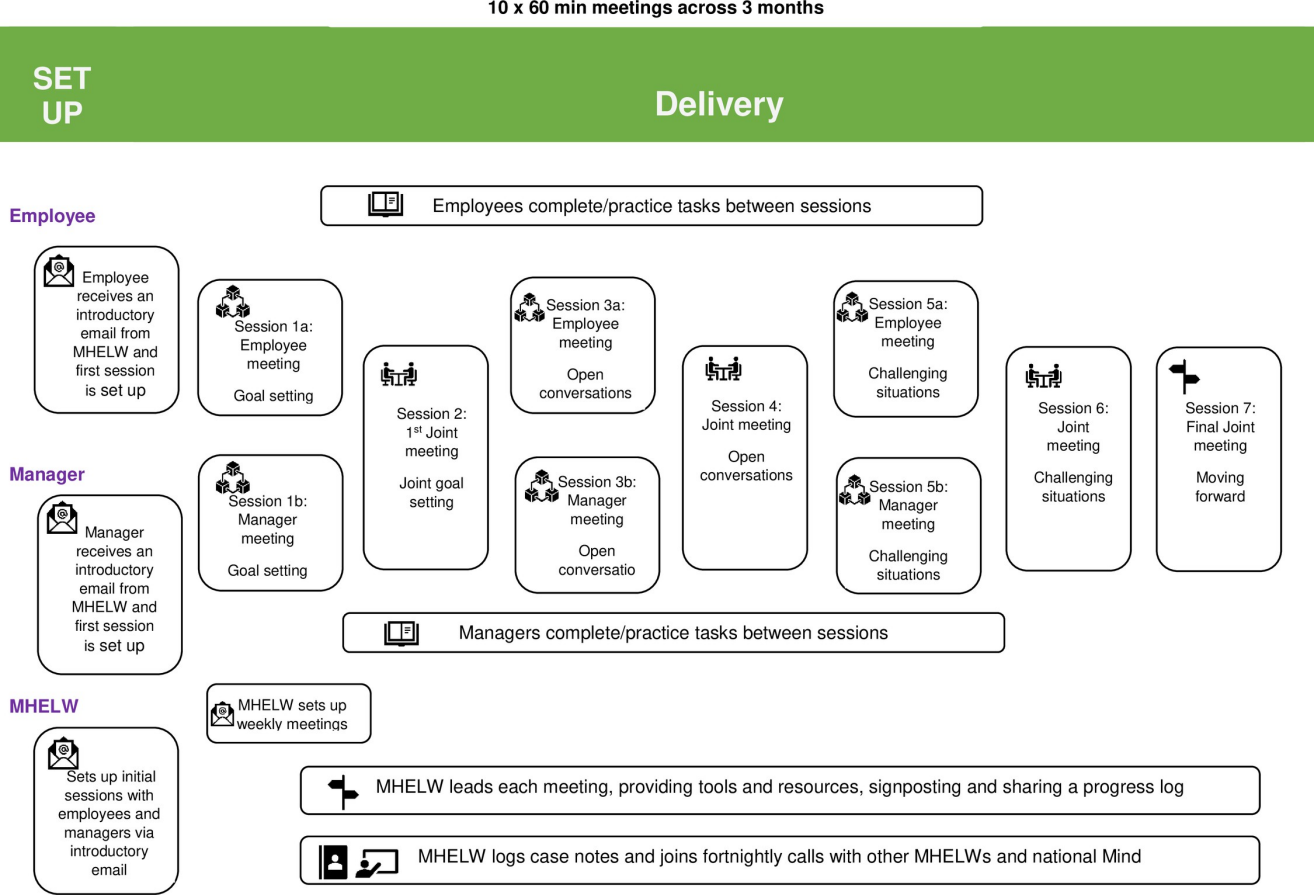
MENTOR intervention overview.

Mental Health Employment Liaison Workers (MHELWs) will hold 10 x 1-hour sessions, with the option of having up to three (out of ten) of the sessions conducted face-to-face, the others on Zoom. MHELWs will be advised to complete delivery of the intervention within three months, there will be flexibility and participants could take longer than three months to complete the programme. Post-intervention questionnaires will be collected once participants complete their last session. Three (out of ten) sessions will be offered to employees only, three (out of ten) sessions to managers only, and four (out of ten) sessions will be held with both parties jointly (Fig 4). Employees and line managers will receive a £10 Amazon voucher upon completion of the baseline and three-month questionnaires.

To ensure that the intervention could be flexibly adapted for delivery during the COVID-19 pandemic and to account for or be flexible to any medium and longer-term changes in working environments, we ensured that the intervention could be provided remotely, although some face-to-face sessions could be facilitated at participant request.

#### Role of the Mental Health Employment Liaison Worker (MHELW)

A priori with mind to scalability, cost and effectiveness of the intervention, we decided that the intervention should be deliverable by a trained worker (who was given two-week MENTOR training programme), though not someone needing formal psychological therapy experience and qualifications. Depending on the size of the organisation, MHELWs will be trained to support several businesses at any one time and be agile with regards to time spent in each business depending on demand. Detailed role descriptors of the MHELW role are available in the (S1 File). The employees and their managers will be allocated the same MHELW. If the MHELW takes sick leave for two-four weeks and/or leaves the job then the MHELW will be replaced with another MHELW.

**Fig 5:**
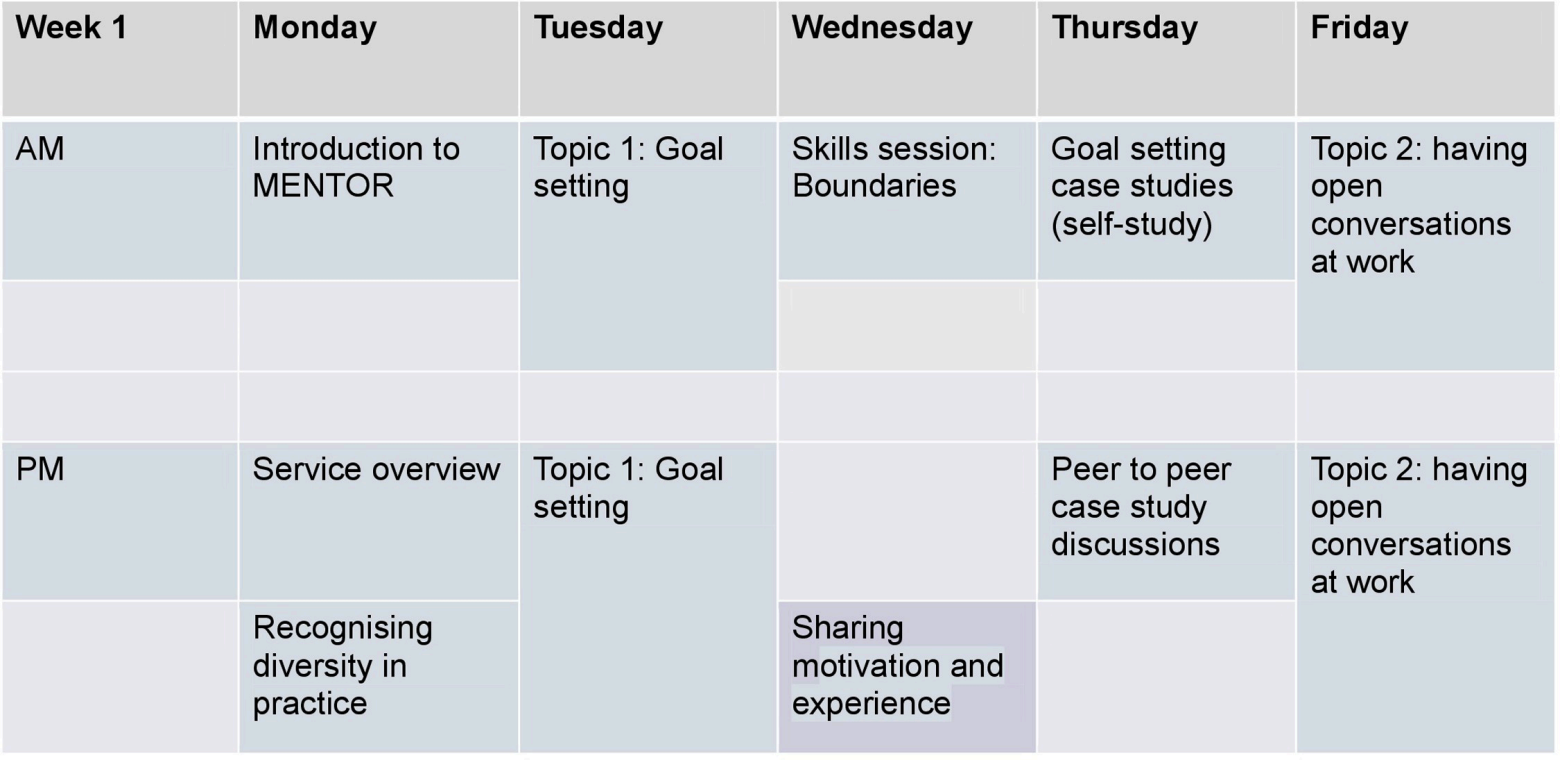
MENTOR training programme.

A training programme was developed by the University of Birmingham research team together with the Mental Health Charity Mind. The training will be delivered by a research fellow with expertise in delivering psychological interventions in the workplace (AP) and the Mental Health Charity Mind and take place in two weeks from 9am to 5pm. Subsequent to the training, there will be ongoing joint supervision of the MHELWs via forums held weekly, as well as a ‘buddy system’ for peer support. See Fig 5 for the structure of the training programme and sessions delivered.

#### MENTOR training programme

The training programme will be conducted remotely (on Zoom) in two weeks with 10 MHELWs to prepare them to deliver the MENTOR intervention. The training will aim to give MHELWs the knowledge and skills to deliver the sessions and information related to the research trial. The training will be delivered by Mind, the University of Birmingham researchers, with facilitation support from the University of Warwick. The training will use: 1) a series of personas based on case studies, 2) icebreakers and breakout groups on Zoom to help build relationships and confidence and eventually mimic MENTOR activities, and 3) an interactive, collaborative tool called Jamboard to allow for live working through exercises, applying knowledge live with virtual post-it notes.

The training will alternate four full-day sessions on the manualised intervention (Topic 1, Topic 2 and Topic 3 and exit plan) with skills sessions (boundaries, working with people, working remotely). The need for the skills sessions was understood in the co-production workshops as important for MHELWs to receive to become confident MHEWLs. An additional session will be delivered on recognising diversity in practice and another session on research perspectives to help MHEWLs understand research aspects and best practices in pilot feasibility trials. Following the main topic sessions on the intervention, MHELWs will be given time during the training to do case studies and attend drop-in sessions for clarity on aspects of the intervention. All sessions will be recorded, and participants will be given access to records. See Fig 5 for a visual representation of MENTOR training schedule.

*Healthcare practitioner role*. The MENTOR intervention was designed with a co-production approach with healthcare practitioners (HCPs), as well as employers and employees. Six interviews with mental HCPs across primary and secondary care in the Midlands who work with people in work were held before designing the programme. These practitioners ranged from Healthy Minds, Improving Access to Psychological Therapies (IAPT) high-intensity Cognitive Behavioural Therapy (CBT), acute inpatient care, community outreach teams and diversion services. They were recruited through MHPP Twitter, wider network channels and a Birmingham and Solihull NHS Mental Health Trust Intranet post.

In each 30-minute interview with the HCPs, we aimed to: 1) Understand whether MENTOR and the HCP involvement felt useful and appropriate to them, 2) Walk through the journey of what participating in the intervention would be like for the HCP and surface any concerns and suggestions, 3) Understand what touchpoints (e.g. initial letter, final report) and information would be useful to have from the MHELW during the intervention (e. g., frequency & format), 4) Understand operational challenges and back office work needed to make this intervention a success and to refine how it will be run.

The results from the interviews indicated that the HCPs thought there was a clear need for this intervention to reduce their busy workload. They reported that having a two-way touchpoint contact with a letter (initial letter from the researchers informing the HCP that the employee was taking part in the intervention) and a final report (researchers sending a final report to the HCP with a summary of achievements reached during the intervention) was an excellent solution to help HCP reduce the busy workload they have to help patients with mental health conditions. The HCPs themselves reported that informing the HCPs concerning the progress of the case (with a final report) could be a good way to reduce healthcare workload. A full-day workshop was given to MHELWs on the writing up of the report to make sure they had all the skills and competencies to write such a report on the completed case.

Participant privacy and consent was discussed with the HCPs during the interviews and with the broader MENTOR team. These discussions were led by a senior Consultant Psychiatrist (SM). It was agreed that the employee needed to be consulted and asked whether the report could be seen and sent to the HCP. According to the suggestions from the HCPs, we decided to incorporate these suggestions into the intervention. Therefore, employees were asked whether they wanted the report to be sent to their HCPs. If the employees agreed to send the report, they also had the opportunity to read it before it was sent.

### Feasibility randomised controlled trial

#### Ethical approval

Ethical approval has been granted by the University of Birmingham’s Science, Technology, Engineering and Mathematics, Research Ethics Committee, Ref: ERN-20-1813 and HRA (IRAS project ID 293809, REC reference: 21/HRA/1913). The study also received University of Birmingham research governance, HRA approvals and separate NHS approvals (Royal Wolverhampton NHS foundation Trust & BSMHFT NHS trust). Sponsorship was granted by the University of Birmingham Governance Team (researchgovernance@contacts.bham.ac.uk).

#### Data management

A detailed data management plan will be enacted with input from the participating business organisations, local Minds (as the service delivery partners) and representatives from the University of Birmingham research data management and legal teams to ensure that our practices and data management procedures are GDPR compliant. The trial does not have a data monitoring committee given the short duration and the low risks associated with MENTOR delivery.

#### Sample size

**Fig 6:**
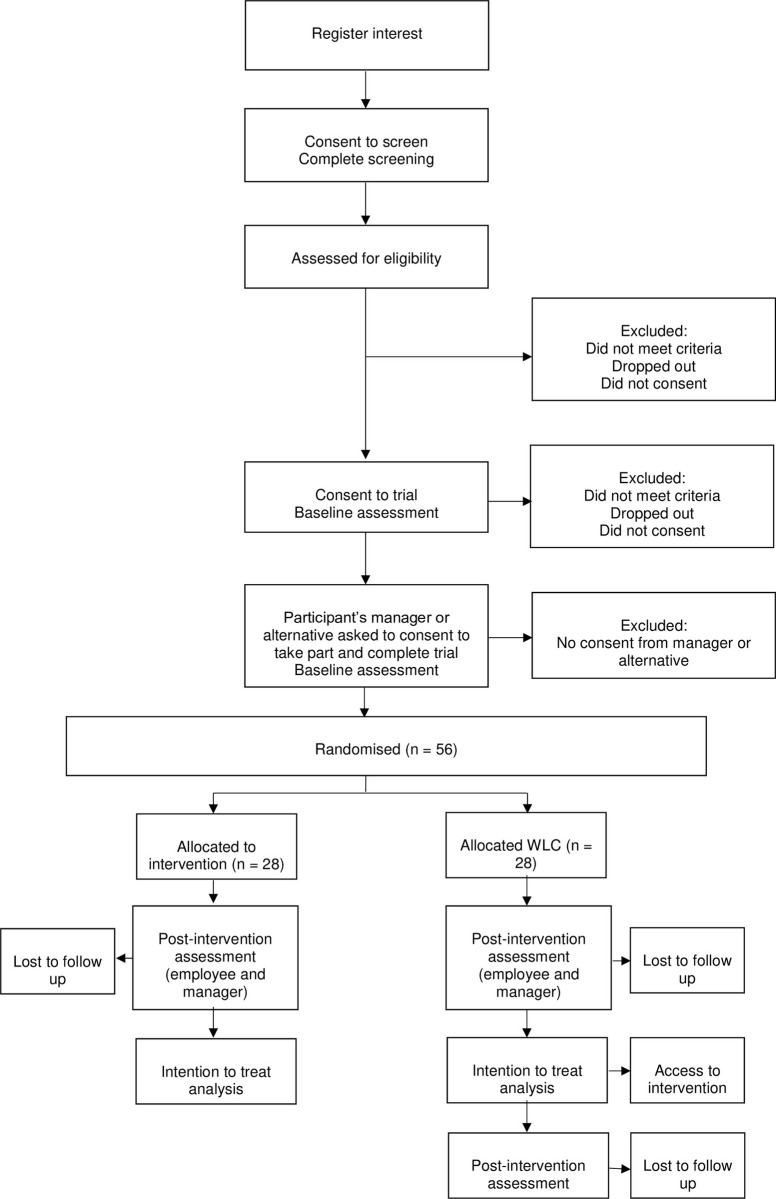
MENTOR trial flow.

There are currently no clear guidelines for estimating appropriate sample sizes for feasibility studies as this is not a fully-powered hypothesis-testing study. Therefore, the sample size is based on pragmatic assumptions around feasible recruitment figures required to estimate the key parameters around the feasibility of a full RCT based [30]. A recommended sample size of between 24 [31] and 50 [32] participants per arm for feasibility RCTs will be used, consistent with the median sample size found in pilot RCTs [33]. Therefore, we aim to recruit 56 employee-manager pairs, who will be randomised into either the intervention or the waitlist control group (28 pairs per arm), factoring in a 25% attrition rate anticipated in a fully powered RCT (Fig 6).

#### Randomisation

Individuals will be randomised to either the intervention or the waitlist control group via a computer-generated 1:1 block randomisation sequence by a statistician independent of the study.

### Recruitment

#### Expression of interest

Awareness of the Mental Health and Productivity Pilot (MHPP) programme will have been raised within organisations and information of the study can be found on the MHPP website (https://mhpp.me/). This will include signposting for employers interested in their organisations taking part in the study. At this stage, we have already received expressions of interest from 33 different organisations, including 16 large (250+employers), 9 medium ((5–250 employees), 6 small (9–49 employees) and 2 very small employers (1–8 employees) (e.g., Universities, NHS trusts, schools, and manufacturing). Businesses will be asked to advertise the study to potential participants (employee and line managers) via emails, direct communications, signs in communal staff areas such as break rooms or canteens, staff bulletins and newsletters. Materials will be provided to employers for this advertisement. We will then gather interest from employees who wish to participate in the study and screen them to identify eligibility.

#### Direct recruitment of employees and line managers

The study will also be advertised to the broader working community, who are not necessarily working for a participating employer, using a variety of local and online recruitment strategies. Advertising and recruitment materials for individuals was conducted through social media networks such as Twitter, Linkedin, Facebook and word of mouth through flyers and posters.

#### Pre-screening phase

After participants register their interest in the study, the research team will contact them with a link (via the Qualtrics platform, Provo, UT) to written consent for an online eligibility screener and complete the screener. The online screener contains questions to ensure participants meet the pre-specified study eligibility criteria as described in the inclusion/exclusion criteria and also collect information regarding the employees’ healthcare professional (HCP). Employees’ HCPs will be notified of their participation in the study and informed that they will receive a report at the end of the intervention.

#### Consenting procedure

There will be three levels of consent for standard recruitment and two levels of consent for direct recruitment. For the standard recruitment route, firstly, we will fully brief employers regarding the nature of the study and then, we anticipate that a main point of contact will be provided by the employer which may include for example the Chief Executive Officer (CEO, an occupational health advisor or a human resource manager. We will then obtain site-level implied consent from the employer to allow their employees to take part in the study. For workers recruited directly, employer onboarding will not be necessary, meaning that implied consent from their employer is not required. We will then check whether employees meet the study’s eligibility criteria and once eligibility is confirmed, individual-level written consent (via the Qualtrics platform, Provo, UT) will be obtained first from employees who agree to take part in the study, and then their line managers for both recruitment routes.

Employees and line managers will both need to consent to take part in the study. Once both consent forms are received by the research team, the MHELWs will initiate contact with them. We will ensure that the identity of the employee is not revealed to the line manager until both the employee and line manager have consented to participate in the study.

Consent form and other relevant documentation will be provided to all study participants (employees and managers). The participants will be given sufficient time to review and understand the information provided before deciding whether to participate. Any questions or concerns regarding the consent form will be addressed by the research team. In addition to the consent form, participants will receive relevant study information sheets and other necessary documentation to ensure they are fully informed about their involvement in the study.

### Outcome measures

Primary and secondary outcome measures will be collected at baseline (pre-intervention) and after three months (post-intervention). Participants in the control group will receive the same intervention after a 3-month delay. Outcome measures will be collected for both employees and managers (see below). All data collection of outcome measures and pre-screening scales are administered using the Qualtrics platform, with data stored on University of Birmingham secure servers in accordance with General Data Protection Regulation (GDPR) and research governance guidelines. A summary of the outcome measures at the various time points is provided in Table 1.

**Table 1:** Data collection process and time-points.

Measured outcome	Data collection method	Assessment method	Measurement time-point		By whom
			Baseline	3 months	
Work productivity	Work Productivity and Activity Impairment: General (WPAI-GH)	Online questionnaire via Qualtrics	✓	✓	Employee (self-report)
Job satisfaction	Indiana Job Satisfaction Scale	Online questionnaire via Qualtrics	✓	✓	Employee (self-report)
Anxiety	General Anxiety Disorder-7	Online questionnaire via Qualtrics	✓	✓	Employee (self-report)
Depression	Patient Health Questionnaire- 9	Online questionnaire via Qualtrics	✓	✓	Employee (self-report)
Health related quality of life	Euro-QOL–five-dimension scale	Online questionnaire via Qualtrics	✓	✓	Employee (self-report)
Sense of control	Sense of control scale	Online questionnaire via Qualtrics	✓	✓	Employee (self-report)
Decisional deficit	Decisional Conflict scale	Online questionnaire via Qualtrics	✓	✓	Employee (self-report)
Days on sick leave	Self-reported employee sickness record	Online questionnaire via Qualtrics	✓	✓	Employee (self-report)
Mental Health Knowledge	Mental health knowledge scale	Online questionnaire via Qualtrics	✓	✓	Line manager (self-report)
Attitude about mental health	Personal depression stigma scale	Online questionnaire via Qualtrics	✓	✓	Line manager (self-report)
Self-efficacy	General self-efficacy scale	Online questionnaire via Qualtrics	✓	✓	Line manager (self-report)
Mental health promotion	Safety promotion intentions scale	Online questionnaire via Qualtrics	✓	✓	Line manager (self-report)
Burnout	Burnout scale	Online questionnaire via Qualtrics	✓	✓	Line manager (self-report)
Work demand	Individual work performance scale	Online questionnaire via Qualtrics	✓	✓	Line manager (self-report)
Number of eligible participants	Trial document (case report form) entry record	Password protected spreadsheet/Qualtrics	✓		Researchers
Number recruited	Trial document (case report form) entry record	Password protected spreadsheet/Qualtrics	✓		Researchers
Number consented	Trial document (case report form) entry record	Password protected spreadsheet/Qualtrics	✓		Researchers
Number retained	Trial document (case report form) entry record	Password protected spreadsheet/Qualtrics		✓	Researchers
Number of sessions completed by the employee	Trial document (case report form) entry record	Password protected spreadsheet/Qualtrics		✓	Record entered by MHELW
Number of sessions completed by the manager	Trial document (case report form) entry record	Password protected spreadsheet/Qualtrics		✓	Record entered by MHELW
Number of weeks spent to complete MENTOR intervention	Trial document (case report form) entry record	Password protected spreadsheet/Qualtrics		✓	Record entered by MHELW
Semi-structured interviews for employees	Face to face/telephone	Audio-recorded		✓	Researchers
Semi-structured interviews for managers	Face to face/telephone	Audio-recorded		✓	Researchers
Focus group (s) with MHELWs	Face to face/telephone	Audio-recorded		✓	Researchers
Intervention evaluation form MHELW		Online questionnaire via Qualtrics		✓	MHELWs

Participants will complete self-reported questionnaire assessments entirely online using the Qualtrics survey platform. They will be informed of their randomisation outcome, whether it is MENTOR or WLC, via email by the trial management team (AP, FJ). Therefore, the participants will not be blind to their treatment allocation, making it a single-blinded trial. The trial management team will not be blind, as they will have access to personal identifiable data but not to the research data, which is all non-identifiable data. Statistical analyses will be carried out by members of the research team (MM, KG), who will be blinded to the allocation and will only have access to non-identifiable research data.

### Primary outcomes: Feasibility and acceptability of MHELW intervention (measured at 3 months) and work productivity

#### 1. Feasibility

- 56 employee-manager pairs of the full RCT sample (with a 25% attrition rate) in a 5-month recruitment period (May to end of September 2022)
- Retention rate of ≥60% employee-manager pairs as measured by providing post-intervention outcome measures attendance
- Estimates of eligible participants recruited, failures to recruit due to recruitment issues and participants dropping out due to feasibility issues
- Completion rate of study questionnaires (employee and line manager) at baseline and 3 months post-enrolment for both intervention and control groups, reported as percentage missing data for each assessment scheduled at baseline and 3 months.

#### 2. Acceptability

- Participants attending ≥ 70% of the sessions (5 out of 7 individual sessions).
- Estimate the rate of agreement/no agreement as to whether the MHELW think in their opinion that each session of the intervention was delivered as intended
- Estimates of failures to recruit due to lack of acceptability, participants dropped out due to lack of acceptability, and adverse or serious adverse events.

#### 3. Work productivity (measured at baseline and 3 months)

Work productivity will be measured by the Work Productivity and Activity Impairment: General Health v2.0 (WPAI: GH) scale [34], a possible primary outcome in a full trial. The WPAI-GH is a 6-item self-report questionnaire consisting of four sub-scales: absenteeism (percentage of work time missed because of one’s health in the past seven days), presenteeism (percentage of impairment experienced while at work in the past seven days because of one’s health), overall work productivity loss (overall work impairment measured by combining absenteeism and presenteeism to determine the total percentage of missed time), and activity impairment (percentage of impairment in daily activities due to one’s health in the past seven days). The WPAI-GH has been shown to demonstrate good internal consistency (α = 0.76) [35] and test-retest reliability (r = 0.71–0.87) [36]. This study will focus on the following three subscales of the WPAI-GH: 1) Presenteeism 2) absenteeism and 3) overall impairment. Higher scores indicate greater impairment [36].

#### Adverse events (both employees and line managers)

The research team will monitor potential adverse events (AE) and serious adverse events (SAE) that may or may not be associated with the treatment via participant self-report measures at each wave of the assessment and through MHELW report. MHELWs will also be following local Mind safeguarding procedures. MHELWs are encouraged to report any AE and SAE at any time throughout the treatment. The research team will develop more detailed definitions of AE and SAE prior to recruitment with input from stakeholders and participating businesses. All AE and SAE will be reported, using a modified version of the un-wanted event to adverse treatment reaction (UE–ATR) checklist for psychological interventions [37].

### Intended secondary outcomes (measured at baseline and 3 months) (employee measures)

#### Job satisfaction

Job satisfaction will be measured using the Indiana Job Satisfaction Scale (IJSS) [38], a brief job satisfaction questionnaire designed for use with individuals with a severe mental illness. The IJSS consists of a 32-item self-report questionnaire, divided into six subscales: ‘General Satisfaction’, ‘Pay’, ‘Advancement and Security’, ‘Supervision’, ‘Co-workers’ and ‘How I feel about this job’ with higher scores indicating greater job satisfaction. The IJSS shows high internal consistency (α = 0.90) and test-retest reliability (r = 0.75) [38].

#### Anxiety

Anxiety is measured using the General Anxiety Disorder-7 (GAD-7) [39]. The GAD-7 is a 7-item self-report questionnaire that is commonly used in primary care and mental health settings as a screening tool and symptom severity measure. Higher GAD-7 scores have been shown to correlate with disability and functional impairment [34]. For example, scores between 10–14 identifies moderate GAD symptoms and describes a possible clinically significant condition whilst scores above 15 indicate severe GAD symptoms and advice for active treatment. The GAD-7 shows good internal consistency (α = 0.79–0.91) [35,36] and test-retest reliability (r = 0.83) [40,41].

#### Depression

The severity of depression is measured using the Patient Health Questionnaire-9 (PHQ-9) [42]. The PHQ-9 is a 9-item self-reported questionnaire that monitors severity of depression and response to treatment. The scale has been validated for use in primary care [43] and has been shown to identify depression in at-risk populations [44]. Higher scores indicate more severe symptoms of depression e.g. scores of 15–19 indicate moderately severe depression (above clinical thresholds) whilst scores of 20–27 indicate severe symptoms (above clinical threshold) [42]. The PHQ-9 shows good internal consistency (α = 0.91) [45] and test-retest reliability (r = 0.81) [46].

#### Health-related quality of life

The health-related quality of life is measured using the Euro-QoL-five-dimensional scale [47]. The EQ-5D 5L is a 6-item self-report outcome measure that evaluates generic quality of life across six subscales: ‘Mobility’, ‘Self-care, ‘Usual activities’, ‘Pain/discomfort’, ‘Anxiety/depression’ and ‘health today. Each dimension has five response levels ranging from no problems (Level 1 to extreme problems (Level 5) [48], the responses are expressed a one digit number that describes one’s health state. Higher numbers indicate poorer quality of life [49]. The EQ-5D-5L shows good internal consistency (α = 0.77) [50] and test-retest reliability (r = 0.73–0.84) [51].

#### Sense of control

It is understood that well-being indexes (for example, depression and health) are associated with a perceived sense of control [52–54]. The Sense of Control scale is a two-dimension scale consisting of a total of 12-items [52]. The Sense of Control scale consists of ‘Personal Mastery’ and ‘Perceived Constraints’. Higher scores on the perceived constraints subscale indicate a greater perceived sense of self control meanwhile higher scores on the personal master subscale indicate higher levels of perceived constraints [55]. The scale demonstrates good internal consistency (α = 0.86 Perceive Constraints; α = 0.70 Personal Mastery) and test-retest reliability (r = 0.78) [56].

#### Decisional conflict

Employee and manager decisional conflict [57] will be measured with the Decisional Conflict Scale (DCS), a 16-item self-report questionnaire designed to measure 5 dimensions of decision making i.e. feeling 1) uncertain, 2) uninformed, 3) unclear about values, 4) unsupported and 5) effective decision making). Scores for each dimension are converted into standardised scores ranging from 0–100 with scores above 37.5 being associated with decision delay [58]. The scale demonstrates good internal consistency (α = 0.79) and test-retest reliability (r = > 0.78) [59].

### Number of days taken on sick leave in the last month (self-reported by employee)

#### Intended secondary outcomes (measured at baseline and 3 months) (line managers)

*Mental Health Knowledge*. Knowledge about mental health will be assessed using the Mental Health Knowledge Schedule (MAKS), a 12-item self-report questionnaire assessing evidence-based knowledge in relation to stigma towards mental health [60]. Higher scores indicate greater mental health knowledge [61]. The scale shows good internal consistency (α = 0.75) [61] and test-retest reliability from 0.57 to 0.87 [62].

#### Personal stigma

Attitudes about mental health will be measured using a modified version of the 9-item self-report Personal Depression Stigma Scale (PDSS) [63]. The PDSS is comprised of two subscales measuring personal and perceived stigma. The personal stigma subscale measures stigma in one’s attitudes towards depression whilst the perceived stigma subscale measures one’s perception about their attitudes of others towards depression [64]. The scale will be modified by replacing “depression” with “mental health problem”. Higher scores indicate higher levels of depression stigma [64]. The scale shows good internal consistency (α = 0.78) and test-retest reliability (r = 0.67–0.71) [64].

#### Self-efficacy

Managers’ mental health self-efficacy will be measured using an adapted version of the 9-item General Self-Efficacy Scale (GSE) which assesses optimistic self-beliefs to cope with various demands in life [65]. Items will be adapted to reflect self-efficacy related to employees’ mental health. The scale ranges from 1 (Strongly Disagree) to 6 (Strongly Agree) and includes the item “I feel confident about promoting employees’ mental health.” Higher scores indicate higher self-efficacy [66]. The scale has good internal consistency (α = 0.76–0.90) [62] and test-retest reliability (r = 0.67) [67].

#### Promotion intentions

Managers’ intentions to promote mental health in the workplace will be measured using an adapted version of a safety scale designed to assess managers’ safety promotion intentions [68]. The measure consists of three items: “It is very likely that I will promote mental health in my workplace,” “I intend to achieve the performance-based goals that I set for myself,” and “I want to apply what I learn about mental health to my work setting” With higher scores indicating greater intentions to promote mental health in the workplace [68].

#### Burnout

The Shirom-Melamed Burnout Measure (SMBM) [69] will be used to measure job-related burnout. This well-established 14-item self-report questionnaire has three subscales designed to capture the burnout syndrome’s core components: physical fatigue, emotional exhaustion, and cognitive weariness. We selected this particular measure for the following reasons: 1) Its theoretical underpinnings are specified, 2) It explicitly seeks to capture a construct that is distinct from depression and anxiety, 3) Unlike more generic burnout measures, responses to the SMBM are temporally anchored to the past 30 workdays. Higher scores indicate greater levels of self-reported burnout however, studies have used an arbitrary cut-off score of ≥4.0 to demonstrate severe burnout [70,71]. The SMBM demonstrates good internal consistency (α = 0.75) [72].

#### Work performance

Work performance will be measured using the Individual Work Performance Questionnaire (IWPQ) [73], an 18-item self-report questionnaire measuring work performance i.e. employee’s behaviours or actions that are relevant to the goals of their organisation [73]. This scale consists of three subscales: task performance, contextual performance and counterproductive work behaviour) with higher scores indicating higher task and contextual performance, and higher counterproductive work behaviour [73]. The IWPQ shows good internal consistency ranging from 0.78–0.85 across the three subscales [73]. A summary of the study measures can be found in Table 1.

### Data analysis plan

Analyses will be performed using SPSS version 27 and statistical software Stata (Stata, version 16.0; Stata Corp). Data analysis will be performed after the last participant has provided their 3-month post-intervention outcome measures. The intention-to-treat (ITT) principle will be applied to the primary analyses with all participants’ data being analysed as per their assigned intervention at baseline. Following randomisation, participants who withdrew consent or those with a protocol violation concerning eligibility will be excluded from the ITT analysis. Participants who do not complete the intervention or are lost to follow-up will be included in the dataset; intention to treat and sensitivity analyses will be performed. Summary statistics will be provided for all variables. Any notes that the MHELW makes will be safely stored in password protected computers and made anonymous.

Descriptive statistics will be used to report on primary and secondary outcome measures. Baseline between-group differences will be tested by conducting independent sample t-tests between the intervention and the control group on outcomes measures.

For the primary outcomes (feasibility and acceptability of the intervention), recruitment and dropout rates will be evaluated using absolute and percentage frequencies. Intervention adherence and engagement will be calculated by assessing the response rate of participants receiving the intervention, response rate of completed questionnaires, and the number of sessions completed by intervention group participants.

For the productivity outcome and the secondary outcomes for employees and managers, a 2 x 2 ANOVA—with Time as the within-subject variable (baseline and 3-month post-intervention) and Condition (MENTOR and control condition) as the between-subject variable–will be conducted to test whether outcome measures improved in the intervention arm relative to the control at the 3-month post-intervention time-point.

### Process evaluation

The process evaluation will use a mixed method approach, combining quantitative and qualitative measures in order to explore engagement, acceptability, barriers, and facilitators, for employees, line managers and MHELWs delivering the intervention.

#### Quantitative data collection

During the course of the study and at 3 months after baseline we will measure, number of sessions completed by the employee, number of sessions completed by the manager and number of weeks spent to complete the MENTOR intervention.

#### Qualitative data collection

Individual semi-structured interviews will be conducted by the researchers with employees and line managers whilst MHELWs will be asked to take part in focus groups to better understand their experiences of receiving and delivering the intervention, respectively. Semi-structured interview schedules will be developed for each stakeholder group in line with the main aims of the study.

Participants who were eligible for the trial and agreed to participate in the interview will be sent a consent form and a participant information sheet for the interviews via email. A purposeful sample of MHELWs will also be invited via email for participation in the focus group interviews post-intervention.

Interviews will be scheduled at a convenient time and location for the interviewee and will be conducted either face-to-face or online as per the interviewee’s preference. All interviews will be audio-recorded on an encrypted device with consent and then subsequently transcribed verbatim. Data processing will guard participant identity. The participants and line managers who have consented and agreed to participate in the qualitative interviews will also receive a £10 voucher.

#### Analysis

Transcribed interview data will be analysed using thematic analysis [74] in NVivo software. We will focus on processes and experiences of how the intervention was delivered, its acceptability, and barriers and enablers of participation in, and engagement with the intervention. We will explore MHELWs’, employees and managers’ views of delivering the intervention via a focus group, including the range of practitioners involved in the intervention delivery. We will explore MHELWs views on the success, impact, mechanisms of action, suitability and ease of delivery of care package components, and any perceived/experienced barriers to delivery at individual, team or systemic/organisational levels. The views of each stakeholder group and comparisons between them will be explored. Within the stakeholder group, we will explore common themes and variations and whether these map onto any sampling variations such as type of business sector, size of firm or region/ local enterprise partnerships (LEPs) areas.

## Discussion

Although several studies have tested the effectiveness of workplace-based programmes to improve employees’ mental health in the workplace, there is a paucity of research showing effectiveness for interventions to improve mental health and productivity for employees with mental health conditions, and their managers. To the best of our knowledge, this will be the first study examining the feasibility and acceptability of an intervention that include both employees, their managers and aim to test the feasibility of a brand new role: a MHELW.

This trial will provide comprehensive data for future intervention developments, inclusive of information relative to the intervention mechanisms of change, and information that will enable an assessment of whether a full trial is likely to be feasible and worthwhile and provide an insight into the parameters it should be designed around. Although it is a feasibility study, the current study will provide important evidence to support alternative support for employees with a mental health diagnosis remaining in work.

Anticipated difficulties for this trial include recruitment of employers and participants during the COVID-19 pandemic as well as during a period of pandemic recovery, retaining employer engagement, and the retention and attrition of participants. Systemic challenges that may arise throughout the course of the study are related to the unpredictable nature of the COVID-19 pandemic and therefore employers not prioritising engagement with research. The COVID-19 pandemic and the associated reduction in face-to-face office working may have impacted the relationship between employees and line managers and make them less inclined to participate because they may not have a close relationship during this time. Additional challenges that may arise with participants’ recruitment include the stringent criteria of solely including employees with a clinical diagnosis of mental health condition(s) and currently receiving professional care from NHS services. Direct recruitment appears to be one solution to recruitment however with the caveat of employees in some cases having to engage with the intervention during working hours. A limitation of this study might be that for pragmatic reasons, measures of anxiety, depression and productivity of employees were preferred for inclusion over self-efficacy (or similar). The intervention was designed to improve employees’ mental health and productivity. For managers, the focus of the intervention was to raise their awareness about mental health problems employees experience at work. In line with the study outcomes for employees–understanding whether MENTOR was a feasible intervention for improving employees’ mental health and productivity–measures were selected accordingly and in line with what was most appropriate to answer the study outcomes.

On the other hand, the early focus on co-production workshops combined with the benefits of academic rigour (researchers from multiple Universities and expertise) alongside user-centric service design (national Mind) and ‘those on the ground’ (local Minds) might have helped to develop an intervention that was more accessible for MHELWs to deliver.

Given the complexity of the intervention spanning a range of mental health conditions and businesses and being delivered by an interdisciplinary team, we believe this pilot will provide essential preliminary data that will inform a fully powered randomised controlled trial. Also, with the impact that the COVID-19 pandemic has had on businesses, we believe that this type of intervention and programme is even more timely than when the study was originally commissioned. If effective, this study will provide preliminary evidence on how we could formally test the intervention to benefit as many workers and businesses as possible.

## Supporting information

S1 Checklist SPIRIT 2013 checklist: Recommended items to address in a clinical trial protocol and related documents. (DOCX)

S1 File Role descriptors of the MHELW. (DOCX)

S2 File Consent form given to employees and managers to take part in MENTOR. (DOCX)

S3 File (PDF)
