# Supplementary material for: Supporting employers and their employees with Mental hEalth problems to remain eNgaged and producTive at wORk (MENTOR): A feasibility randomised controlled trial protocol
Source: PLoS One. 2023 Apr 20;18(4):e0283598. doi: 10.1371/journal.pone.0283598 (PMC10118171; doi:10.1371/journal.pone.0283598)
Supplement: S1 File — (DOCX) [file pone.0283598.s002.docx]

**Mental Health and Productivity Pilot**

**MENTOR – Draft Protocol**

| ***Study title*** | *Supporting employers and their employees with* ***M****ental h****E****alth problems to remain e****N****gaged and produc****T****ive at w****OR****k (MENTOR): A feasibility randomised controlled trial of a Mental Health Employment Liaison Worker (MHELW) intervention* |
| --- | --- |

**August 2021**

Version 6

(31.08.2021)

**IRAS ID: 293809**

Table of Contents

Introduction3

Background/aims3

Methodology6

Data analysis plan24

Process evaluation25

Ethical considerations29

References 30

1. **Introduction**

|  |  |
| --- | --- |

1. **Background and aims**

People with mental health problems find it very hard to find jobs and remain in work [1-3]. Approximately one sixth of workers experience a mental health problem at any one time [4] and anxiety and depression are considered to be responsible for almost half of working days lost in Britain due to health problems [5]. Whilst the total cost to businesses of mental health problems at work is close to £45 billion, the bulk of this is due to presenteeism at approximately £30 billion [5], more than three times the cost of employee absence. Presenteeism is defined as attending work whilst ill and therefore not performing at full ability. It can be both positive and negative and be due to a variety of factors. Compared to people with physical health problems, people with depression who are employed are much more likely to be unemployed at 6 months, or experience high rates of absenteeism, presenteeism or job turnover [6]. This is one reason why the mental health problems are so costly to individuals, employers, the economy and society.

Whilst models such as Individual Placement and Support (IPS) are effective at helping people to obtain work, they tend to be less successful at helping people keep it and are largely focussed on people with severe mental health problems such as Schizophrenia [7, 8]. The largest proportion of employees with mental health problems have mood disorders such as depression, rather than schizophrenia. Their needs and employer views and processes for this group are likely to be different.

The UK Government also has an “Access to Work” programme, which is able to provide grants and also mental health support to people about to start work or in work. Whilst not based in businesses, an Access To Work representative will visit a workplace to assess the person’s need and recommend adaptations and support that can be provided. They do not operate at the interface between businesses and workers, aiming to improve mental health and productivity outcomes [9].

Furthermore, the impact of the COVID-19 pandemic on mental health is a very serious concern. It is predicted that approximately 41.8% of the UK population are at high risk of mental health problems because of the economic vulnerability and negative economic shock as a direct result of the COVID-19 outbreak [10]. Additionally, a recent report by Durcan and colleagues [11], estimated that should the UK experience a recession effect similar to that of the 2008 banking crisis, at least 500,000 more people could experience mental health problems if the economic impact of the pandemic mirrors those of the last recession.

Currently, this is a space where there is an unmet need, and a lack of evidence about what may help. That is, individuals who have been unwell and are receiving some form of treatment already (such as GP contact, Improving Access to Psychological Therapy (IAPT), third sector counselling or input from secondary mental health services) often do not have the support at work to remain engaged and productive in their employed role. This may be particularly the case in Small and Medium sized Enterprises (SMEs) which may not have occupational health departments to rely on to advise and support workers with mental health problems. Furthermore, not all employees would feel comfortable disclosing unmanageable stress or poor mental health to their employers [12].

Businesses and employees may not know how to tackle the presenteeism related to mental health problem, responses need to be framed by the legal framework, there may be conflict between the parties, as well as consequences to work colleagues. One of the largest wellness studies conducted in the UK exploring the link between modifiable health risks and short-term productivity found that the UK economy lost approximately £92 billion as a result of ill-health related absences and presenteeism in 2019 from factors such as depression, poor lifestyle and stress. The study also revealed that British businesses lose on average 37 working days per employee per year due to physical and mental health related absences and presenteeism in 2019. The study revealed a rise in presenteeism with approximately 45% of UK employees admitting suffering from presenteeism in 2019, a two third rise (24%) from 2014. Young workers aged (18 to 25 years) were particularly vulnerable with 55% reporting unable to perform at their peak productivity, compared to 38% of employees aged 45 or over [13]. Traditional healthcare providers alternatively may not have enough knowledge of job roles or business structures to be able to help employers and employees alike adequately and effectively.

In order to address this intervention and evidence ‘gap’ and building on discussions between the partners within the wider research team (including with clinicians and mental health service providers) we propose to innovate and assign a specifically trained Mental Health Employment Liaison Worker to work at the interface between employers, employees and mental health providers. We will test the feasibility and acceptability of this intervention thoroughly as this is a new innovative model meeting a need that is currently poorly served, but potentially critical to improving the wellbeing and mental health of workers. Feasibility and pilot studies are conducted before a main study to address areas of uncertainty that will enable a successful full definitive trial [14] This feasibility pilot study aims to examine the feasibility, acceptability and preliminary estimates of a 3-month Mental Health Employment Liaison Worker (MHELW) intervention in supporting the mental health and productivity of employees with a clinical diagnosis of mental health problems who are working.

**Research Question:**

To test whether the Mental Health Employment Liaison Worker (MHELW) intervention is acceptable and feasible in supporting employees with a clinical diagnosis of mental disorder and currently receiving treatment by an NHS healthcare provider?

1. **Methodology**

Study design: A feasibility randomised controlled trial with a 3-month MHELW intervention supporting workers with a clinical diagnosis of mental disorder to remain engaged and productive compared to control, receiving the intervention after a 3-month delay. The trial will be registered on the ISRCTN registry.

**Population:** Adult workers with a clinical diagnosis of a mental health disorder, who are currently receiving treatment by an NHS healthcare provider.

***Inclusion criteria:***

- Employees with a clinical diagnosis of a mental health disorder
- Employees currently receiving care for mental health problems through NHS services
- Aged ≥18 years
- Able to give written informed consent
- Fluent in English
- Willing to work with the MHELW and their business line manager

***Exclusion criteria:***

- Currently in acute mental health crisis as defined by their clinical team
- Currently on extended sick leave (i.e., >4 weeks)
- Receiving input from an Individual Placement and Support (IPS) Worker
- Planning to retire within the next 10 months and unable to complete intervention and evaluation

**Intervention:** Employment focussed support and input from a Mental Health Employment Liaison Worker (MHELW). The reporting of the intervention will be aligned to the Template for Intervention Description and Replication (TIDier) checklist [15], to ensure that MHELW are consistent and transparent in reporting/documenting each of the 10 intervention sessions across the 3 months study intervention. The MHELW will provide support to employees identified as having clinically significant, mental health problems (i.e., those who are currently receiving statutory NHS treatment), to remain engaged and productive at work. The role of the MHELW will be to liaise between employers, employees and care providers so that businesses and employees can receive dedicated and optimised support and advice on what good workplace wellbeing practices look like for each individual and how stakeholders can help achieve this. This will facilitate the process via which employees can continue to work, and employers can be helped to make this happen, likely reducing the probability that employees then are absent from work.

Depending on the size of the organisation, a MHELW will support several businesses at any one time and be agile on time spent in each business depending on demand. Each trial participant will be supported by an MHELW for a period of three months. A three-month period was selected as it prioritises dynamic facilitation and action in the short term to have an impact and increases the likelihood that the intervention will be cost-effective. Due to the outbreak of COVID-19, MHELWs will provide support both face-to-face and online, due to ongoing home working and social distancing requirements during the pandemic and beyond the operation of the MHELW will need to be implemented across both forms of delivery. The MHELW will not provide formal (e.g., manualised) psychological therapy for employees.

MHELWs will hold 10x1hr sessions across the 3 months, with the option of 3 of these conducted face to face. Then sessions will be made of from: 3 employees only, 3 managers only and 4 joints with both parties. The MHELW is present to identify the accommodations from both employer and employee that could be made for the employee to continue being supported and to continue at work. Employees/line manager will receive a £10 voucher at the end of the study intervention. Further, some participants and line managers who have consented and agreed to take part in the qualitative interview, will also receive an additional £10 voucher. Additionally, the intervention will be free to participant and environmentally friendly, convenient to access, will not require workers to take time off work, and will not require extra travel/costs.

**Pre-screening phase**

After participants register their interest in the study, the research team will contact participants with a link to consent for an online eligibility screener. The online screener contains specific questions to ensure participant fits the study eligibility criteria as described in the inclusion/exclusion. After a short online screener to determine eligibility, employees will be enrolled in the study, however they will have an equal chance of ending up in the waitlist control or the intervention group. Capacity limitations may mean that not all eligible employees will be invited to participate. Employee’s healthcare professionals (HCP) will be notified of their participation in the study. Furthermore, HCP’s will be asked to provide some information about the impact of the intervention on employee’s job and health and wellbeing.

**Withdrawal of Study Participants**

A participant may voluntarily withdraw participation in this study at any time. If a participant chooses to withdraw from the study following consent, either the research fellows (FJ and AP) or the lead researcher (SM) will delete their personal data (name and email address) from the dataset. The data will be deleted within 5 working days of the request, and the participant will be informed of the confirmation of removal of their personal data and withdrawal from the study. However, data which has already been collected and anonymised after the final follow-up data collection of the trial cannot be withdrawn as participants cannot be identified. Withdrawal emails will be acknowledged, and confirmation sent to the participant by the research fellows. The participant will be asked if they wish to delete their data and whether there was any reason they withdrew from the study, but do not have to respond should they not wish to provide a reason. The email will then be deleted immediately, and the research team will be informed of the withdrawal and request of data deletion. ID authentication will be conducted, and data deletion will be conducted under supervision of the lead researcher to confirm deletion.

**Role descriptors of the MHELW:**

These are based in part on the Individual Placement Support (IPS) employment specialists and the IPS fidelity scale [16], but with modifications and specifications aimed at supporting people already in employment to maintain this and improve their well-being. These have been developed and refined by Mind (the provider organisation) subsequent to internal Mind stakeholder consultation groups. These descriptors are:

1. An ethos that recovery is likely and therefore work can be maintained

1. Zero exclusion – a person’s desire to continue to be employed is the only criterion for access to the MHELW.
2. The support provided will be based on employee preferences
3. The MHELW will work independent to the business and employee but will focus on both sets of needs-this is based on the idea that it is in the interests of both the business and employee that the individual be supported to continue to work, and their experience of work be improved
4. The support to the business and employee will be individually tailored but will in general terms involve stage 1 (preparatory meetings with both parties where process and desired outcomes are explored), stage 2 (involving tailored support regarding adaptations, education, mental health support), and stage 3 (review of actions and impacts and future planning)
5. Acting as main point of liaison between NHS providers, the business (line manager) and employees subject to consents. In practice this may involve attending joint meetings between the employee and manager or holding individual meetings and advising on what adaptations might be necessary for the worker and supporting these to happen. It may also involve providing feedback to care providers in the form of a report on progress made and topics covered during the intervention, as well as background on the participant’s workplace context (subject to consent).
6. from the business (subject to consent)
7. Monitoring and reviewing adaptations
8. Upskilling the business line manager and employees about the importance of work for mental health by providing relevant mental health education
9. Providing basic mental health support to employees, but not any formal therapy as this might interfere with their current NHS treatment plan
10. High accessibility of help-links with employer platforms, posters, emails sent to staff
11. MHELWs will have initial group training and ongoing group supervision
12. Maximum number of people per MHELW will be 20 (similar to IPS programmes)
13. If one is available, the MHELW will also work with and develop relationships with the occupational department of the business
14. The MHELW will have contact with healthcare providers (subject to consent). Employers will not have access to health records or letters.

During the trial preparation critical components (e.g., training manual) will be manualised to aid implementation and future replication, but also used as a basis to develop the training programme. The intervention will last for a maximum of 3 months-the point at which primary outcome measurements will be taken.

**MHELW training/recruitment**

We will seek to recruit individual with a minimum of 6 months experience of providing mental health and wellbeing services and have experience in providing advice, information and support about workplace wellbeing and positive mental health at work. MHELW will be trained (jointly with research team members) and employed by the charity Mind (or local Minds), who have taken leadership in defining and developing the role via their established service design and development process. A training programme will be developed and delivered to the MHELWs as a coherent group, so that individuals can learn together and the group can test out ideas, define the boundaries of the intervention and further develop it. The training will take place over the course of two weeks. Subsequent to the training, there will be ongoing joint supervision of the MHELW via meetings held at least monthly, as well as a ‘buddy system’ for peer support. The MHELWs will also have line managers for support within local Minds. Aspects that the manual and training will cover includes (but not limited to):

1. What MHELW can or cannot do
2. Very strong role of consent
3. Adherence to medication
4. Interaction with legal framework
5. Competencies
6. Minimum and maximum number of sessions

**Control group**

Participants eligible for this intervention who are not randomised to the intervention arm, will be entered into a waiting list control arm. The control arm will receive the same intervention after a 3-month delay.

**Randomisation**

Individuals will be randomised to the intervention and the control group. Although stratification at the organisational level by size (large, medium, or small) and location (LEPs) is not practical in this study, these organisational characteristics along with demographic (age, gender, SES) and work environment characteristics (industry, job nature) will be included in our exploratory analyses as covariates. Randomisation will be carried out via a computer-generated random allocation sequence by a researcher independent to the study.

**Full** **randomised controlled trial sample size**

There have been no previous randomised controlled trial (RCT) studies reporting effect sizes of liaison workers intervention programmes on work productivity. With limited empirical findings to base our sample size calculations on, we estimate a small to medium effect size of (d=0.33). The estimated sample size necessary to detect an effect size (Cohen’s d) of 0.33 at an alpha error rate of 0.05 (two-tailed) and a beta error rate of 0.20 using the G* Power 3 program was 292 participants (146 participants per arm) [17-18]. However, we anticipate we may need to obtain consent from up to 365 participants at randomisation, anticipating a 25% attrition rate. This equates to a N point difference between the arms in our primary outcome measure.

**Feasibility randomised controlled trial sample size**

There are currently no clear guidelines for estimating an appropriate sample size for feasibility studies. This is not a hypothesis testing study and therefore the sample size is based on pragmatic assumptions around feasible recruitment figures and the number of participants required to estimate the key parameters around the feasibility of a full RCT based on the sample size calculation. As per standard practice in feasibility RCTs, no formal power calculation is required [14]. A recommended sample size between 24 [19] and 50 [20] participants per arm for feasibility RCTs will be used, consistent with the median sample size found in pilot RCTs [21]. We aim to recruit 56 participants (15% of the full RCT sample size) to the whole study, who will be randomised into either the intervention or the waitlist control group (28 participants per arm) factoring a 25% attrition rate as describe above for the full RCT.

**Recruitment**

**Expression of interest employee**

Awareness of the Mental Health Productivity Pilot (MHPP) programme will have been raised within organisations (via initiatives such as the Thrive Plus programme) and expression of interest though the Mental Health Productivity Pilot website (<https://mhpp.me/>). We will recruit businesses to be part of the study through the network that we have already established during proposal scoping and the earlier studies. They will all be given the opportunity to take part subject to local service delivery partner availability. We have already received expressions

of interest from widely different businesses. Businesses will be asked to advertise the study to potential participants (employee and line managers) via emails, direct communications, signs in communal staff areas such as break rooms or canteens, staff bulletins and newsletters. Once partners have signed up, we will gather interest from employees who wish to take part in the study and screen them to identify eligibility. We will also be advertising the study directly to employees in the wider Midlands community.

**Direct recruitment of employees and line managers**

The study will be advertised to the wider working community, who are not employees of partners, using a variety of local and online recruitment strategies. Advertising and recruitment materials for individuals are additional to the materials used for recruiting employees through employers. All advertising materials for individual recruitment include a link to a mirroring version of the study website, intended for these employees

Advertising to individuals may include:

- Advertisement in the local newspapers and wider community locations (parks; supermarkets; town halls; community halls)?
- The study will be also be advertised using various social media platforms which may include LinkedIn, Twitter, Facebook, Instagram, Reddit, and any other relevant social media platforms identified by the research team.

Participating NHS trusts will be requested to advertise the study via their Communication team who will be provided with poster and newsletter which will direct participants to the study website. We will request that NHS trusts advertise the study as widely as possible. The study researchers will also hold online presentations and webinars to businesses to engage them and their employees/line managers. We will ask employers from across the Midlands to register interest in the trial in their commitment to support their employees/line manager. The role of the employer acts as a gatekeeper to the prospective participants, the employees and line manager, who we wish to recruit. Any employers that are interested can meet with the research team who will contact explain the study in more detail and outline the minimum commitment expected by employers (e.g., providing resources such as computer and internet, allowing employees to complete intervention related activities during normal work hours). Once an employer registers with the study, we will ask employers to advertise our study via different internal channels to their staff. Interested employees and line manager will be able to register their voluntary interest in the study by contacting the research team directly without needing to speak to their employer. The research team will contact participants (employees and line managers) with a link to consent for an online eligibility screener. The research team will then contact eligible employees and line manager by sending them a participant information sheet and asking them to complete a consent form (mirroring versions for employees of partner employers and individuals) if wishing to participate. Individuals recruited from the wider community may not have employers acting as a gatekeeper, therefore there may not agreement that they will be allowed to complete the trial “MHELW sessions” during working hours. Because this is different for individuals who have not been recruited through a partner, there will be separate mirroring PIS’s and consent forms for MENTOR.

**Consenting procedure**

There will be 3 levels of consent. Firstly, we will obtain site-level consent from the employer to host the study (implied consent will be taken if we are given access to the business). Individual-level consent will be obtained from managers involved in managing individuals with a clinical diagnosis of mental health problems and workers who agree to take part in the study. Participants will be initially asked to express an interest in taking part in the study, after which they will be invited to consent and complete the screening questionnaires and subsequently complete the eligibility screening questionnaire. There will be two mirroring versions of the expression of interest questionnaire: one for employees recruited via partner employers and one for individuals whose employer has not signed up and who have been directly approached. The expression of interest for individuals from the latter route will additionally be asked for their workplace address to ensure they are in the Midlands based workforce and to ensure our local Mind can deliver the study sessions. To ensure unintended disclosures do not occur as a result of this process, workers who are approached in this way will be explicitly made aware that their consent will mean that we approach their named line managers also for the study. We will also advertise the study in the workplace who expressed an interest in the study and provide contact details for employees/line managers to take part.

We will ensure that the identity of the employee is not revealed to the line manager until both have consented to participate. Employers will be fully briefed about the study and we would anticipate that a senior manager within the business will be allocated by the business to be our main point of contact. This may be e.g., CEO, senior manager, occupational health advisor, human resources manager, etc. We will also provide information and support which the employers will receive in preparation for the study. Prior to finalisation, the nature, content and feasibility of this input will be discussed with a number of business partners to ensure their perspectives are fully taken into account. Only if an employee and a line manager both consent to take part in the study will we proceed, as the MHELW requires study contact with both.

**Study setting**

The interventions will be conducted in the workplace and online, that is, the intervention site is the business. Working individuals who do not have partner employers acting as gatekeepers are free to decide when they participate in the study. However, non-partner employers are not obliged to allow participation during working hours, and this is made clear on the working individual consent form versions.

**Outcomes: Data collection**

The main outcome measures of the full RCT will be used. In addition to assessing the fidelity of the intervention with respect to key outcome measure scores at 3 months assessments will be completed at baseline and 3 months (at intervention end). Each participant (intervention and control group) will be involved in the study for a maximum of 3 months in total at which point primary and secondary outcome data will be collected at baseline and 3 months in both allocation arms. Participants in the control group will receive the same intervention after a 3-month delay, as part of service delivery by Mind, but no further outcome data will be collected from them. Since the line managers will have some of the study interventions, we will explore how the intervention impacted line managers mental health awareness, line management skills and job demand. All data collection of outcome measures and pre-screening scales are administered using the Qualtrics platform, with data stored on the Qualtrics servers in accordance with GDPR governance. A summary of the outcome measures at the various time points is provided in table 1.

***Feasibility and acceptability of MHELW intervention (measured at 3 months).***

**Feasibility:**

- Recruitment of 15% (n=55) employees of the full RCT sample size (n=365 with 25% attrition rate) in a 5-month recruitment period (May to end of September 2021)
- Retention rate of ≥60% as measured by attendance at the post intervention assessment
- Estimates of eligible participants recruited, failures to recruit due to recruitment issues and participants dropping out due to feasibility issues
- Completion rate of study questionnaires (employee and line manager) at baseline and 3 months for both intervention and control groups, reported as percentage missing data for each assessment schedule at baseline and 3 months.

**Acceptability:**

- Participants attending ≥ 70% of the sessions (5 out of 7 individual sessions).
- Estimate the rate of agreement/no agreement as to whether the MHELW think in their opinion that each session of the intervention was delivered as intended
- Estimates of failures to recruit due to lack of acceptability, participants dropped out due to lack of acceptability, and adverse or serious adverse events.

***All assessment schedules intended for use in the definitive trial will be used in this feasibility pilot study. These are:***

***Intended primary outcome: work productivity (measured at baseline and 3 months)***

This will be measured by the Work Productivity and Activity Impairment: General Health v2.0

(WPAI:GH) scale [22]. The WPAI-GH consists of 4 metrics: absenteeism (the percentage of work time missed because of one’s health in the past 7 days), presenteeism (the percentage of impairment experienced while at work in the past 7 days because of one’s health), overall work productivity loss (overall work impairment measured by combining absenteeism and presenteeism to determine the total percentage of missed time), and activity impairment (the percentage of impairment in daily activities because of one’s health in the past 7 days). The WPAI has been shown to demonstrate good internal consistency. This study will focus on the work productivity items of the WPAI-GH. Presenteeism (percentage of impairment experienced while at work in the past seven days due to health problems), absenteeism (percentage of work time missed due to health problems in the past seven days), overall impairment (combination of absenteeism and presenteeism). The WPAI-GH outcomes are expressed as impairment percentages, with higher numbers indicating greater impairment and less productivity. The WPAIGH contains the following six questions with a recall period of the last week: Q1=currently employed; Q2=hours missed due to health problems; Q3=hours missed due to other reasons; Q4=hours actually worked; Q5=degree health affected productivity while working; and Q6=degree health affected regular activities. Absenteeism is defined as the percentage of time absent from work due to health of the last week and is calculated by the formula Q2/(Q2+Q4) ×100%. Presenteeism is measured by the degree health problems affected work productivity of the last 7 days on a rating scale ranging from 0 to 10, with 0 indicating that health problems had no effect on my work and 10 indicating that health problems completely prevented me from working. The outcome is expressed as a percentage score representing the impairment due to health reasons while working, with higher numbers indicating greater impairment and less productivity, and is calculated by the formula (Q5/10) ×100%.

***Intended secondary outcomes (measured at baseline and 3 months): Employee***

1. **Job satisfaction**

Measured using the Indiana Job Satisfaction Scale which is a brief job satisfaction questionnaire designed for use with individuals with a severe mental illness. The IJSS consists of a 32-item self-report questionnaire, divided into six subscales: ‘*General Satisfaction’*, ‘*Pay*’, ‘*Advancement and Security’*, ‘*Supervision*’, ‘*Co-workers’* and ‘*How I feel about this job’*. The IJSS shows high internal consistency (α = 0.90) and test-retest reliability (r = 0.75) [23].

1. **Anxiety**

Anxiety is measured using the General Anxiety Disorder-7 [24]. The GAD-7 is commonly used in primary care and mental health settings as a screening tool and symptom severity measure. Using the threshold score of 10, the GAD-7 has a sensitivity of 89% and specificity of 82%, with high test-retest reliability (ICC = 0.83). Higher GAD-7 scores have been shown to correlate with disability and functional impairment [25]. A score of 5-9 indicates only mild GAD and suggests monitoring these individuals, 10-14 identifies moderate GAD symptoms and describes a possible clinically significant condition, whilst scores above 15 indicate severe GAD symptoms and advises for active treatment.

1. **Depression**

The severity of depression is measured using the Patient Health Questionnaire-9 [26]. The PHQ-9 assesses the severity of depression across the nine DSM-IV criteria on a 0-3 Likert scale. The scale has been validated for use in primary care [27]. The PHQ-9 has been shown to identify depression in at risk populations [28]. A criterion score of ≥10 has been shown to have an 88% and a specificity of 88% for major depression [26]. Scores of 0-4 denote no clinically significant symptoms; scores between 5-9 denote subthreshold symptoms; scores of 10-14 indicate moderate symptoms (below clinical thresholds); scores of 15-19 indicate moderately severe (above clinical thresholds) whilst scores of 20-27 indicate severe symptoms (above clinical threshold).

1. **Health-related quality of life**

The health-related quality of life is measured using the Euro-QoL-five-dimensional scale [29]. The EQ-5D 5L consists of 6 items stemming across: ‘Mobility’, ‘Self-care’, ‘Usual activities’, ‘Pain / discomfort’, ‘Anxiety / depression’ and ‘health today’. The scale has shown to demonstrate reliable test-retest effects (ICC > 0.7), and significant concurrent validity (rs ≥ 0.44).

1. **Sense of control**

It is understood that well-being indexes (for example depression and health) are associated with a perceived sense of control [30-32]. The Sense of Control scale is a two-dimension scale consisting of a total of 12 items [30]. The Sense of Control scale consists of ‘Personal Mastery’ and ‘Perceived Constraints’. The scale demonstrates good internal consistency (α = 0.86 Perceive Constraints; α = 0.70 Personal Mastery).

1. **Decisional conflict**

Employee and manager decisional conflict [33]: Decisional Conflict Scale: a 16-item scale designed to measure personal perceptions of uncertainty in choosing between options, modifiable factors contributing to uncertainty including feeling uninformed, lack of clarity about personal values and feeling unsupported in decision making, effective decision making such as feeling the choice is informed, values-based, likely to be implemented and expressing satisfaction with the choice

1. **Days taken on sick leave in the last month (self-reported by employee)**

***Intended secondary outcomes (measured at baseline and 3 months): Line Manager***

1. **Mental Health Knowledge.**

Knowledge about mental health will be assessed using the 12-item Mental Health Knowledge Schedule [34]. The scale of the MAKS ranges from 1 (Strongly Agree) to 5 (Strongly Disagree); an example item is “Most people with mental health problems want to have a paid employment.” The scale shows good item retest reliability from 0.57 to 0.87.

1. **Personal stigma.**

Attitudes about mental health will be measured using a modified version of the 9-item Personal Depression Stigma Scale [35]. The scale will be modified by replacing the word “depression” with the term “mental health problem.” The scale ranges from 1 (Strongly Agree) to 5 (Strongly Disagree). The scale shows good internal consistency (α = 0.76).

1. **Self-efficacy**

Managers’ self-efficacy surrounding mental health will be measured using an adapted version of the 9-item General Self-Efficacy Scale [36]. Items will be adapted to reflect self-efficacy related to employee mental health. The scale ranges from 1 (Strongly Disagree) to 6 (Strongly Agree) and includes the item “I feel confident about promoting employee mental health.” Reliability ranges from 0.76 to 0.90.

1. **Promotion intentions.**

Managers’ intentions to promote mental health in the workplace will be measured using an adapted version of a safety scale designed to assess managers’ safety promotion intentions [37]. The measure consists of three items: “It is very likely that I will promote mental health in my workplace,” “I intend to achieve the performance-based goals that I set for myself,” and “I want to apply what I learn about mental health to my work setting.” The scale ranged from 1 (Strongly Disagree) to 6 (Strongly Agree).

1. **Burnout.**

The Shirom-Melamed Burnout Measure [38] will be used to measure job-related burnout. This well-established 14-item scale has three subscales that are designed to capture core components of the burnout syndrome: physical fatigue, emotional exhaustion, and cognitive weariness. We selected this particular measure for the following reasons: (1) its theoretical underpinnings are clearly specified; (2) it explicitly seeks to capture a construct that is distinct from depression and anxiety; and (3) unlike more generic burnout measures, responses to the SMBM are temporally anchored to the past 30 workdays.

1. **Work performance.**

Work performance will be measured with the IWPQ [39]. This scale consists of three scales: task performance, contextual performance and counterproductive work behaviour) with a total of 18 items. Each subscale ranges between 0 and 4.

**Table 1: Data collection process and timepoint**

| **Measured outcome** | **Data collection method** | **Assessment method** | **Measurement timepoint** | | **By whom** |
| --- | --- | --- | --- | --- | --- |
|  |  |  | **Baseline** | **3 months** |  |
| Work productivity | Work Productivity and Activity Impairment: General (WPAI-GH) | Online questionnaire via Qualtrics | 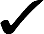 | 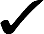 | Employee (self-report) |
| Job satisfaction | Indiana Job Satisfaction Scale | Online questionnaire via Qualtrics | 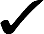 | 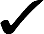 | Employee (self-report) |
| Anxiety | General Anxiety Disorder-7 | Online questionnaire via Qualtrics | 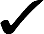 | 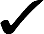 | Employee (self-report) |
| Depression | Patient Health Questionnaire- 9 | Online questionnaire via Qualtrics | 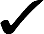 | 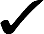 | Employee (self-report) |
| Health related quality of life | Euro-QOL – five-dimension scale | Online questionnaire via Qualtrics | 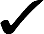 | 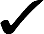 | Employee (self-report) |
| Sense of control | Sense of control scale | Online questionnaire via Qualtrics | 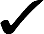 | 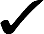 | Employee (self-report) |
| Decisional deficit | Decisional Conflict scale | Online questionnaire via Qualtrics | 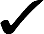 | 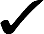 | Employee (self-report) |
| Days on sick leave | Self-reported employee sickness record | Survey via Qualtrics | 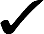 | 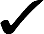 | Employee (self-report) |
| Mental Health Knowledge | Mental health knowledge scale | Online questionnaire via Qualtrics | 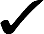 | 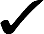 | Line manager (self-report) |
| Attitude about mental health | Personal depression stigma scale | Online questionnaire via Qualtrics | 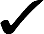 | 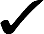 | Line manager (self-report) |
| Self-efficacy | General self-efficacy scale | Online questionnaire via Qualtrics | 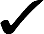 | 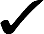 | Line manager (self-report) |
| Mental health promotion | Safety promotion intentions scale | Online questionnaire via Qualtrics | 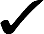 | 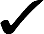 | Line manager (self-report) |
| Burnout | Burnout scale | Online questionnaire via Qualtrics | 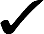 | 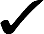 | Line manager (self-report) |
| Work demand | Individual work performance scale | Online questionnaire via Qualtrics | 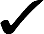 | 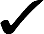 | Line manager (self-report) |
| Number of eligible participants | Trial document (case report form) entry record | Password protected spreadsheet/Qualtrics | 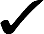 |  | Research Fellow |
| Number recruited | Trial document (case report form) entry record | Password protected spreadsheet/Qualtrics | 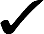 |  | Research Fellow |
| Number consented | Trial document (case report form) entry record | Password protected spreadsheet/Qualtrics | 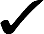 |  | Research Fellow |
| Number retained | Trial document (case report form) entry record | Password protected spreadsheet/Qualtrics |  | 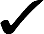 | Research Fellow |
| Number of contacts of MHELW | Trial document (case report form) entry record | Password protected spreadsheet/Qualtrics |  | 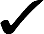 | Record entered by MHELW |
| Length of contact of MHELW | Trial document (case report form) entry record | Password protected spreadsheet/Qualtrics |  | 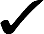 | Record entered by MHELW |
| Number of contacts  between MHELW and NHS providers | Trial document (case report form) entry record | Password protected spreadsheet/Qualtrics |  | 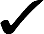 | Record entered by MHELW |
| Referrals to non- statutory providers | Trial document (case report form) entry record | Password protected spreadsheet/Qualtrics |  | 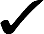 | Record entered by MHELW |
| Semi-structured interviews | Face to face/telephone | Audio-recorded |  | 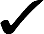 | Research Fellow |

1. **Data analysis plan**

Data analysis will be performed after the last trial participant has completed their 3-month post-intervention. Descriptive statistics (means, SDs, and medians) will be used to report recruitment, retention rates and efficacy outcomes measures (both primary and secondary). Baseline between-group differences will be tested by conducting independent sample t-tests between the intervention group and the control on outcomes measures. A 2 x 2 ANOVA - with Time as the within-subject variable (baseline and 3-month post-intervention) and Condition (MENTOR and control condition) as the between-subject variable – will be conducted to test whether outcome measures improved in the active condition relative to the control at the 3-month post-intervention time-point. Intervention adherence will be calculated by assessing the response rate of participants receiving the intervention, response rate of completed questionnaires, and the number of sessions completed by intervention group participants. Recruitment and dropout rates will be evaluated using absolute and percentage frequencies. Analyses will be performed using statistical software Stata (Stata, version. 16.0; Stata Corp) and SPSS version 24. The intention-to-treat (ITT) principle will be applied to the primary analyses with all participants’ data being analysed as per their assigned intervention at baseline. Following randomisation participants who withdrew consent or those with a protocol violation concerning eligibility will be excluded from the ITT analysis. Participants with missing baseline information and those who do not complete intervention or are lost to follow-up will be included in the dataset; multiple data imputation and sensitivity analyses will be performed. Summary statistics will be provided for all variables.

Data will be collected using online proforma, the pre-screen questionnaire will be administered using Qualtrics survey platform. All data collection of outcome measures and pre-screening scales are administered using the Qualtrics platform, with data stored on the Qualtrics servers in accordance with GDPR governance. Any notes that the MHELW makes will be uploaded and made anonymous in the same way. MHELW will be provided access to the Qualtrics platform using a unique identification code via the email address MHELW provides with a chosen password to upload any intervention notes.

1. **Process Evaluation**

The process evaluation will use a mixed method approach and use the RE-AIM (reach, effectiveness, adoption, implementation, and maintenance) framework [40]. It will be made up of two parts:

1. **Quantitative data collection:**

During the course of the study and at 3 months after baseline we will measure

- Number of contacts with MHELW
- Length of contacts of MHELW
- Number of contacts between MHELW and NHS providers
- Referrals to non-statutory providers

1. **Qualitative data collection:**

We will conduct a qualitative evaluation aiming to explore engagement, acceptability, barriers, and facilitators, for employees, line managers and MHELWs delivering the intervention. Participants who were selected and agreed to take part in the interview will be sent a consent form and participant information sheet via email. We will explore what have MHELW been asked to do, whether they feel able to complete these actions and what they have felt unskilled for. Qualitative data will be collected from sub- samples of these 3 stakeholder groups using either individual interviews or focus groups depending on acceptability to businesses and workers. These are:

1. Employees (N=16-20) individual interviews,
2. Line Managers (N=16-20) individual interviews
3. MHELWs (N=6-8)-focus group.

Participants will be purposively sampled to take account of sector, job role, seniority, age, gender, and those who completed the intervention. Three separate semi-structured interview schedules will be developed focused on exploring the main aims of the qualitative study. Some schedule components are likely to overlap. Interviews will be scheduled at a convenient time and location for the interviewee. Interviews will be audio-recorded on an encrypted device with consent and then subsequently transcribed verbatim. Any references and names to participants will be redacted using search and replace functions, all references to names will be replaced with randomly generated pseudonyms using <https://www.fakenamegenerator.com>. Recordings will be transferred from University of Warwick to the Research Data Store (RDS) on the University of Birmingham servers and deleted immediately from the recording device once this is safely transferred. The audio files will then be deleted once transcription is completed. A professional transcription service will assist in the transcription of the interviews. The transcriber will sign a Confidentiality Agreement and will comply with the data protection act. The anonymised interview transcript will be deleted after 10 years from RDS on the University of Birmingham servers. Interviews will be conducted face-to-face or by telephone/online. Microsoft Teams will be used to provide a video conference platform for the online interviews. However, due to the current COVID-19 pandemic and social distancing measures interviews may be conducted remotely (telephone/online). Furthermore, a purposeful sample of MHELWs will be invited via email to consider participating in a focus group interview at the end of the 3 months intervention to ensure a broad range of participant experiences is captured. Once, the MHELWs expressed an interest in participating, the same recruitment procedures will be followed, including sending an information sheet and a consent form to participate via email. It is possible that discussion about certain topic could be sensitive and upsetting that may results in distress or discomfort. It is possible that participants could disclose information requiring action. To minimise risks we will ensure that, (a) MHELWs receive adequate training in how to support participants and manage their discussions in a constructive manner; (b) any disclosures requiring action are passed on to the research team for action. It will be made clear to participants that they may withdraw consent at any point; (c) Participants are aware that they can choose to omit answering questions. Also, participants are free to withdraw from the trial at any time should they wish to on account of perceived intrusion/inconvenience.

**Analysis**

Transcribed data will be analysed using thematic analysis [41] in NVivo software. We will focus on processes and experiences of how the intervention was delivered, its acceptability, as well as barriers and enablers of participation in, and engagement with, the intervention. We will explore MHELW’s views of delivering the intervention via a focus group including the range of practitioners involved in intervention delivery. We will explore practitioner views on the success, impact, mechanism of action, suitability and ease of delivery of care package components, as well as any perceived/ experienced barriers to delivery at individual, team or systemic/organisational levels. The views of each stakeholder group, and comparisons between them will be explored. Within stakeholder group we will explore common themes and variations, and whether these map onto any of the sampling variations such as type of business sector, size of firm or region/LEP areas.

**Data security/storage**

All trial (personal and research) data will be stored on the University of Birmingham server. Access to personal data is limited to the trial management team, the CI and the Research Fellows (FJ and AP). Study data will be stored only with pseudo-anonymous ID variable and kept separate from personal data accessed by the University of Birmingham research team. Study data and personally identifiable data collected in this study will be stored in password protected files stored on the University of Birmingham Research Data Store (RDS) and back-up following UoB back-up and retention policy. All personal data is stored in a master file, separate from the outcome data collected as part of the study. The master file and the outcome data will only be accessible by the CI and the research fellow (FJ and AP). The master file will also contain participants pseudo-anonymised study IDs. The master file will exist as a password protected *.xlsx file on RDS of the University of Birmingham servers. A separate password protected file consisting only of contact data (Name, email address and phone number and postal address) without pseudo-anonymised study IDs, will be accessible to UoB trial management team (CI, FJ and AP) stored on RDS of the University of Birmingham servers. Access is required to send out any emails such as reminders or request to complete questionnaires. Study and personal data will be deleted from the Qualtrics platform by one the research fellows (FJ, AP), immediately after being transferred to the Research Data Store (RDS) on the University of Birmingham servers. We will request deletion of all personal data from RDS on the University of Birmingham servers immediately after the trial is completed (anticipated date: 02/2022). The pseudo-anonymised study data and anonymised interview transcript will be deleted after 10 years from RDS on the University of Birmingham servers. Participants will be requested to agree to their pseudo-anonymised research data being used in future research which has ethics approval.

**End of Trial Procedures**

At the end of the 3 months intervention, once primary outcome data has been obtained, employees who were in the waiting list control group will receive the active intervention. Those in the intervention group will not receive any further trial specific intervention but some will take part in the process evaluation. We have taken this step (consistent with the pragmatic design) so that all businesses and workers involved in the programme can potentially benefit, especially given the current and possible future Covid-19 situation. Once we have analysed the results, we will publish them in peer-reviewed medical or healthcare journals. We will also publish our results on our Mental Health Productivity Pilot (MHPP) website (<https://mhpp.me/>) and the study findings will also be summarised in the final study report. If participants request it, they will be sent a copy of the published findings. Furthermore, a sustainability plan will be drawn up by the MHELW with the employee and line managers, in order to maintain beneficial changes and to continue to review and make progress against goals beyond the end of the service.

1. **Ethical consideration**

A detailed data management plan will be drafted with input from the participating business organisations, local Minds (as the service delivery partners) and representatives from the university’s research data management and legal teams to ensure that our practices and data management procedures are GDPR compliant. Screening will be delivered online on Qualtrics Participants will be provided with access using a unique participant identification code with their chosen passwords. No identifiable information (e.g., GPS location or IP addresses) will be used for analysis nor be stored in the system for longer than necessary. We will obtain ethics and regulatory approvals, as necessary.

**Adverse events**

Potential adverse effects (AE) and serious adverse effects (SAE) that may or may not associated with the treatment will be monitored by the research team, via participant self-report, at each wave of assessment and MHELW report. Participants are encouraged to report any AE and SAE any time throughout the treatment. More detailed definitions of AE and SAE will be developed by the research team with inputs from stakeholders and participating businesses. All AE and SAE will be reported, using a modified version of the [42] un-wanted event to adverse treatment reaction (UE–ATR) checklist for psychological interventions.

1. **References**
2. Lloyd-Evans B, et al. The nature and correlates of paid and unpaid work among service users of London Community Mental Health Teams. Epidemiology and psychiatric sciences, 2013. 22(2): p. 169-180.
3. Marwaha S, Durrani A, and Singh S. Employment outcomes in people with bipolar disorder: a systematic review. Acta Psychiatrica Scandinavica, 2013. 128(3): p. 179- 193.
4. Marwaha, S. and S. Johnson. Schizophrenia and employment. Social psychiatry and psychiatric epidemiology, 2004. 39(5): p. 337-349.
5. McManus S, et al. Mental health and wellbeing in England: Adult Psychiatric Morbidity Survey 2014. A survey carried out for NHS Digital by NatCen Social Research and the Department of Health Sciences, University of Leicester. 2016.
6. Deloitte: Mental health and employers Refreshing the case for investment [https://www2.deloitte.com/uk/en/pages/consulting/articles/mental-health-and-](https://www2.deloitte.com/uk/en/pages/consulting/articles/mental-health-and-employers-refreshing-the-case-for-investment.html..html) [employers-refreshing-the-case-for-investment.html..html](https://www2.deloitte.com/uk/en/pages/consulting/articles/mental-health-and-employers-refreshing-the-case-for-investment.html..html). 2020 [accessed 29^th^ Sep 2020].
7. Lerner D, et al. Unemployment, job retention, and productivity loss among employees with depression. Psychiatric Services, 2004. 55(12): p. 1371-1378.
8. Bond G.R, Drake RE, Becker DR. Generalizability of the Individual Placement and Support (IPS) model of supported employment outside the US. World psychiatry, 2012. 11(1): p. 32-39.
9. Knapp M, et al. Supported employment: Cost‐effectiveness across six European sites. World Psychiatry, 2013. 12(1): p. 60-68.
10. Department of Works and Pension. Access to Work: factsheet for customers. [https://www.gov.uk/government/publications/access-to-work-factsheet/access-to-](https://www.gov.uk/government/publications/access-to-work-factsheet/access-to-work-factsheet-for-customers) [work-factsheet-for-customers](https://www.gov.uk/government/publications/access-to-work-factsheet/access-to-work-factsheet-for-customers). 2020 [accessed 29^th^ Sep 2020].
11. Mental Health Foundation. The Covid-19 pandemic financial inequality and mental health: a briefing from the coronavirus mental health in the pandemic study. [https://www.mentalhealth.org.uk/sites/default/files/MHF-covid-19-inequality-mental-](https://www.mentalhealth.org.uk/sites/default/files/MHF-covid-19-inequality-mental-health-briefing.pdf) [health-briefing.pdf.](https://www.mentalhealth.org.uk/sites/default/files/MHF-covid-19-inequality-mental-health-briefing.pdf) 2020 [accessed 29^th^ Sep 2020].
12. Durcan G, O’Shea N, Allwood L. Covid-19, and the nation’s mental health. Centre for Mental Health. [https://www.centreformentalhealth.org.uk/sites/default/files/2020-](https://www.centreformentalhealth.org.uk/sites/default/files/2020-05/CentreforMentalHealth_COVID_MH_Forecasting_May20.pdf) [05/CentreforMentalHealth_COVID_MH_Forecasting_May20.pdf](https://www.centreformentalhealth.org.uk/sites/default/files/2020-05/CentreforMentalHealth_COVID_MH_Forecasting_May20.pdf). 2020. [accessed 29^th^ Sep 2020].
13. CIPD. (2015) Getting under the skin of workplace conflict: tracing the experiences of employees [online]. Survey report. London: Chartered Institute of Personnel and Development. [https://www.cipd.co.uk/Images/getting-under-skin-workplace-](https://www.cipd.co.uk/Images/getting-under-skin-workplace-conflict_2015-tracing-experiences-employees_tcm18-10800.pdf) [conflict_2015-tracing-experiences-employees_tcm18-10800.pdf](https://www.cipd.co.uk/Images/getting-under-skin-workplace-conflict_2015-tracing-experiences-employees_tcm18-10800.pdf) [accessed 10^th^ Oct 2020]
14. Vitality Health Study – health at work. [https://www.vitality.co.uk/media-online/britains-healthiest-](https://www.vitality.co.uk/media-online/britains-healthiest-workplace/pdf/2019/health-at-work-2019_uk.pdf?la=en&hash=E87840347CDEFAA86C144EDF16C6053243F490AA) [workplace/pdf/2019/health-at-work-](https://www.vitality.co.uk/media-online/britains-healthiest-workplace/pdf/2019/health-at-work-2019_uk.pdf?la=en&hash=E87840347CDEFAA86C144EDF16C6053243F490AA) [2019_uk.pdf?la=en&hash=E87840347CDEFAA86C144EDF16C6053243F490AA.](https://www.vitality.co.uk/media-online/britains-healthiest-workplace/pdf/2019/health-at-work-2019_uk.pdf?la=en&hash=E87840347CDEFAA86C144EDF16C6053243F490AA) [accessed 14th Oct 2020].
15. Arain M, Campbell MJ, Cooper CL, Lancaster GA. What is a pilot or feasibility study? A review of current practice and editorial policy, BMC Med Res Methodol, 2010. 10(67): p.2-7
16. Hoffmann TC, et al. Better reporting of interventions: template for intervention description and replication (TIDieR) checklist and guide. BMJ, 2014. 348: p.1687.
17. Bond GR, et al. A fidelity scale for the individual placement and support model of supported employment*.* Rehabilitation Counseling Bulletin, 1997. 40: p. 265-284.
18. Faul F, et al. G*Power 3: A flexible statistical power analysis for the social, behavioural, and biomedical sciences. Behaviour Research Methods, 2007*.* 39: p.175- 191.
19. Faul F, et al. Statistical power analyses using G*Power 3.1: Tests for correlation and regression analyses. Behaviour Research Methods*,* 2009. 41: p.1149-1160.
20. Julious SA. Sample size of 12 per group rule of thumb for a pilot study. Pharmaceut Stat, 2005, 4: p.287-291
21. Sim J, Lewis M. The size of a pilot study for a clinical trial should be calculated in relation to considerations of precision and efficacy. J Clin Epidemiol, 2012. 65: p.301-308
22. Billingham SAM, Whitehead AL, Julious SA. An audit of sample sizes for pilot and feasibility trials being undertaken in the United Kingdom registered in the United Kingdom Clinical Research Network database. BMC Med Res Methodol. 2013, 13: p. 104-110
23. Reilly MC, Zbrozek AS, Dukes EM. The validity and reproducibility of a work productivity and activity impairment instrument, 1993. 4(5): pp.353-65
24. Resnick SG, Bond GR. The Indiana Job Satisfaction Scale: job satisfaction in vocational rehabilitation for people with severe mental illness. Psychiatr Rehabil J, 2001. 25(1): 12-9
25. Spitzer RL, Kroenke K, Williams JBW, Löwe B. A brief measure for assessing generalized anxiety disorder: the GAD-7. Arch Intern Med, 2006. 166(10): 1092-7.
26. Ruiz MA, Zamorano E, García-Campayo J, Pardo A, Freire O, Rejas J. Validity of the GAD-7 scale as an outcome measure of disability in patients with generalized anxiety disorders in primary care. J Affect Discord, 2011. 28(3): 277-86.
27. Kronke K, Spitzer R, Williams JBW. The PHQ-9: Validity of a brief depression severity measure. J Gen Intern Med, 2001. 16(9): 606-613.
28. Cameron IM, Crawford JR, Lawton K, Reid IC. Psychometric comparison of PHQ-9 and HADS for measuring depression severity in primary care. Br J Gen Pract. 2008, 58(546): 32-6.
29. Haddad M, Walters P, Phillips R, Tsakok J, Williams P, Mann A, Tylee A. Detecting Depression in Patients with Coronary Heart Disease: a Diagnostic Evaluation of the PHQ-9 and HADS-D in Primary Care, Findings From the UPBEAT-UK Study. PLoS One, 2013. 8(10): e78493.
30. Rabin R, Gudex C, Selai C, Herdman M. From translation to version management: a history and review of methods for the cultural adaptation of the EuroQol five- dimensional questionnaire. Value Health, 2014. 17(1): 70-76
31. Lachman ME, Weaver SL. The sense of control as a moderator of social class differences in health and well-being. J Per Soc Psychol, 1998. 74(3): 763-773.
32. Sanders A, Spenser A. Why do poor adults rate their oral health poorly? Aust Dent J, 2005. 50(3): 161-167.
33. Ward MM. Sense of control and self-reported health in a population-based sample of older Americans: Assessment of potential confounding by affect, personality, and social support. Int J Behav Med, 2013. 20(1): 140-147.
34. O'Connor A. User manual - decisional conflict scale. Ottawa: Ottawa Hospital Research Institute. <https://decisionaid.ohri.ca/docs/develop/User_Manuals/UM_Decisional_Conflict.pdf>. 2010 [accessed 29^th^ Sep 2020].
35. Lacko SE, Little K, Meltzer H, Rose D, Rhydderch D, Henderson C, Thornicroft G. Development and psychometric properties of the mental health knowledge schedule. Can J Psych, 2010. 55(7): p.440-8.
36. Griffiths M, Christensen H, Joum AF, Evans K, Groves C. Effect of web-based depression literacy and cognitive behavioural therapy interventions on stigmatizing attitudes to depression: randomised controlled trial. By J Psych, 2008. 185:342-349.
37. Chen G, Gully SM, Eden D. Validation of a new general self-efficacy scales. Org Res Meth. 2001, 4:62-83
38. Mellen JE, Kelloway EK. Safety leadership. A longitudinal study of the effects of transformational leadership on safety outcome. J Occ Org Psych. 2009, 82: p.253-272
39. Shirom A, Melamed S. A comparison of the construct validity of two burnout measures in two groups of professionals. Int J S Man. 2006, 13: p.176-200
40. Koopmans L, Coffeng JK, Bernaards CM, Boot CRL, Hildebrandt VH, de Vet HCW, Van Der Beek AJ. Responsiveness of the individual work performance questionnaire. BMC Pub Health. 2014, 513:2-11
41. Glasgow RE, Vogt TM, Boles SM. Evaluating the public health impact of health promotion interventions: the RE-AIM framework. Am J Public Health, 1999. 89(9):1322–7.
42. Pope C, Ziebland S, Mays N, Qualitative research in health care: Analysing qualitative data. BMJ, 2000. 320(7227): p. 114.
43. Linden M. How to define, find and classify side effects in psychotherapy: From unwanted events to adverse treatment reactions. Clin Psychol Psychtherap. 20 (4): 286- 296.
