# Supplementary material for: Supporting employers and their employees with Mental hEalth problems to remain eNgaged and producTive at wORk (MENTOR): A feasibility randomised controlled trial protocol
Source: PLoS One. 2023 Apr 20;18(4):e0283598. doi: 10.1371/journal.pone.0283598 (PMC10118171; doi:10.1371/journal.pone.0283598)
Supplement: S2 File — (DOCX) [file pone.0283598.s003.docx]

| **Study Title:** | *Supporting employers and their employees with* ***M****ental h****E****alth problems to remain e****N****gaged and produc****T****ive at w****OR****k (MENTOR)* |
| --- | --- |

CONSENT FORM - Employee

**Participant ID:** *If applicable*

**Principal Investigator: Professor Steven Marwaha**

Please initial all boxes

1. I have read the information sheet (version 1.3, 02.03.21) concerning my participation in this study and have had the opportunity to discuss and ask questions. All my questions have been answered in a satisfactory way and I give my consent voluntarily to participate in this study.
2. I understand that my participation is voluntary and that I am free to withdraw at any time without giving any reason, and that my future care and management will not be affected. If you withdraw from the study, it will often not be possible to withdraw your data which has already been collected and anonymised after the final follow-up data collection of the trial.
3. I understand that information about me, where relevant to the study, will be stored by the research team at the University of Birmingham. Some of this information may be accessed by our collaborator, the mental health charity Mind and local Minds in order to deliver the intervention.
4. I understand that personnel from the University of Birmingham research team may share my personal information with related NHS bodies and NHS regulatory authorities and may access my records for health-related research purposes (even after my incapacity or death).
5. (If appropriate) I agree to my Healthcare Professionals being informed of my participation in the study

1. I understand that I may be contacted in the future for related research purposes and that my data to be used in future research
2. I agree to take part in the above study.

|  |
| --- |
| Name of participant |

|  |
| --- |
| Date |

|  |
| --- |
| Signature |

|  |
| --- |
| Signature |

|  |
| --- |
| Name of person taking consent |

|  |
| --- |
| Date |

| **Study Title:** | *Supporting employers and their employees with* ***M****ental h****E****alth problems to remain e****N****gaged and produc****T****ive at w****OR****k (MENTOR)* |
| --- | --- |

CONSENT FORM – Line Manager

**Participant ID:** *If applicable*

**Principal Investigator: Professor Steven Marwaha**

Please initial all boxes

1. I have read the information sheet (version 1.3, 02.03.21) concerning my participation in this study and have had the opportunity to discuss and ask questions. All my questions have been answered in a satisfactory way and I give my consent voluntarily to participate in this study.
2. I understand that my participation is voluntary and that I am free to withdraw at any time without giving any reason, and my job and legal right not affected. If you withdraw from the study, it will often not be possible to withdraw your data which has already been collected and anonymised after the final follow-up data collection of the trial.
3. I understand that information about me, where relevant to the study, will be stored by the research team at the University of Birmingham. Some of this information may be accessed by our collaborator, the mental health charity Mind and local Minds in order to deliver the intervention.
4. I understand that personnel from the University of Birmingham research team may share my personal information with related NHS bodies and NHS regulatory authorities and may access my records for health-related research purposes (even after my incapacity or death).
5. I understand that I may be contacted in the future for related research purposes and that my data to be used in future research
6. I agree to take part in the above study.

| Name of Participant | Date |  | Signature |
| --- | --- | --- | --- |
| Name of Person taking consent  Line Manager consent form | Date |  | Signature  v1.3 03.03.21 |

| **Study Title:** | *Supporting employers and their employees with* ***M****ental h****E****alth problems to remain e****N****gaged and produc****T****ive at w****OR****k (MENTOR)* |
| --- | --- |

CONSENT FORM – EMPLOYEE (PROCESS EVALUATION)

**Participant ID:** *If applicable*

**Principal Investigator: Professor Steven Marwaha**

Please initial all boxes

1. I confirm that I have read and understood information sheet (version 1.3, 02.03.21). I have had the opportunity to consider the information, ask questions, and have these answered satisfactorily.
2. I understand that my participation in an interview is voluntary and I am free to withdraw at any time, without giving a reason and without my employment or legal rights being affected. I understand that information collected during the interview will be included in the study after being anonymised. If you withdraw from the study, it will often not be possible to withdraw your data which has already been collected and anonymised after the final follow-up data collection of the trial.
3. I understand that the information collected will be used for the study purpose only and that I will not be identified in any way in the analysis and reporting of the results.
4. I agree to the interview being audio-recorded and understand that the recordings will kept secure at the University of Birmingham, and that everything I say will be kept confidential in accordance with the General Data Protection Regulations 2018.
5. I understand that the transcription of the audio recording will be done by a specialist transcription company and will be handled in accordance with the General Data Protection Regulations 2018.
6. Data collected that identifies me by name, (consent form), will be transferred from where it is collected, anonymised, and stored at the University of Birmingham. I agree to the transfer and storage of this data.
7. I agree that verbatim quotes from the interview can be used anonymously in any publication of the research findings

1. I agree that quotes from the interview can be used anonymously in any publication of the research findings
2. I am happy for my data to be used in future research.
3. I agree to take part in the above study
4. (If appropriate) I agree to my Healthcare Professional being informed of my participation in the study

Name of Participant Date Signature

Name of Person Date Signature taking consent

| **Study Title:** | *Supporting employers and their employees with* ***M****ental h****E****alth problems to remain e****N****gaged and produc****T****ive at w****OR****k* ***(MENTOR)*** |
| --- | --- |

CONSENT FORM – LINE MANAGER (PROCESS EVALUATION)

**Participant ID:** *If applicable*

**Principal Investigator: Professor Steven Marwaha**

Please initial all boxes

1. I have read the information sheet (version 1.3, 02.03.21) concerning my participation in this study and have had the opportunity to discuss and ask questions. All my questions have been answered in a satisfactory way and I give my consent voluntarily to participate in this study.
2. I am involved in this study solely on a voluntary basis and I am being asked to participate in an interview as part of a research study. If you decide to participate in the study now, you may change your mind at any time to stop participating in the study. If you withdraw from the study, it will often not be possible to withdraw your data which has already been collected and anonymised after the final follow-up data collection of the trial.
3. I have read and understood the purpose of this study and why it is being carried out
4. I understand that the information collected will be used for the study purpose only and that I will not be identified in any way in the analysis and reporting of the results.
5. I understand the risk of participating in this study is minimal because no confidential information or company policy will be discussed
6. I agree to the interview being audio-recorded and understand that the recordings will kept secure at the University of Birmingham, and that

everything I say will be kept confidential in accordance with the General Data Protection Regulations 2018.

1. I understand that the transcription of the audio recording will be done by a professional transcription company and will be handled in accordance with the General Data Protection Regulations 2018.
2. Data collected that identifies me by name, (consent form), will be transferred from where it is collected, and stored at the University of Birmingham. I agree to the transfer and storage of this data.
3. I agree that verbatim quotes from the interview can be used anonymously in any publication of the research findings
4. I agree that quotes from the interview can be used anonymously in any publication of the research findings
5. I am happy for my data to be used in future research.
6. I agree to take part in the above study

Name of Participant Date Signature

Name of Person Date Signature taking consent

**Consent form (Mental Health Employment Liaison Worker – Focus group)**

| **Study Title:** | *Supporting employers and their employees with* ***M****ental h****E****alth problems to remain e****N****gaged and produc****T****ive at w****OR****k (MENTOR)* |
| --- | --- |

**Participant ID**: If applicable

**Principal Investigator**: Professor Steven Marwaha Please initial all boxes

| 1. I have read the information sheet (version 1.0, 02.03.21) concerning my participation in this study and have had the opportunity to discuss and ask questions. All my questions have been answered in a satisfactory way and I give my consent voluntarily to participate in this study. | | |  |
| --- | --- | --- | --- |
| 1. I am involved in this study solely on a voluntary basis and I am being asked to participate in a focus group as part of a research study. If you decide to participate in the study now, you may change your mind at any time to stop participating in the study. | | |  |
| 1. I have read and understood the purpose of this study and why it is being carried out | | |  |
| 1. I understand that the information collected will be used for the study purpose only and that I will not be identified in any way in the analysis and reporting of the results. | | |  |
| 1. I understand the risk of participating in this study is minimal because no confidential information or company policy will be discussed | | |  |
| 1. I agree to the focus group being audio-recorded and understand that the recordings will be kept secure at the University of Birmingham, and that everything I say will be kept confidential in accordance with the General Data Protection Regulations 2018. | | |  |
| 1. I understand that the transcription of the audio recording will be done by a professional transcription company and will be handled in accordance with the General Data Protection Regulations 2018. | | |  |
| 1. Data collected that identifies me by name, (consent form), will be transferred from where it is collected, and stored at the University of Birmingham. I agree to the transfer and storage of this data. | | |  |
| 1. I agree that verbatim quotes from the interview can be used anonymously in any publication of the research findings | | |  |
| 1. I agree that quotes from the interview can be used anonymously in any publication of the research findings | | |  |
| 1. I am happy for my data to be used in future research. | | |  |
| 1. I agree to take part in the above study | | |  |
| -----------------------------------------  Name of Participant | ----------------------------------  Date | --------------------------------------  Signature | |
|  |  |  | |
|  |  |  | |
|  |  |  | |
| -----------------------------------------  Name of person taking consent | ----------------------------------  Date | --------------------------------------  Signature | |

**Consent form v.1.3**

**Participant Identification Number for this study:**

| **Study Title:** | **INWORK**: INterventions to improve mental health in the WORKplace: A pilot study |
| --- | --- |
| **Investigator(s):** | Arianna Prudenzi (University of Birmingham), Steven Marwaha (University of Birmingham), Feroz Jadhakhan (University of Birmingham), Krishane Patel (University of Warwick), Talar Moukhtarian (University of Warwick), Carla Toro (University of Warwick), Laura Chandler (University of Warwick), Nicole Tang (University of Warwick), Lukasz Walasek (University of Warwick), Caroline Meyer (University of Warwick) |

Please initial all boxes

1. I confirm that I have read and understand the information sheet (INWORK v1.4, 15/01/2021) for the above study. I have had the opportunity to consider the information, ask questions and have had these answered satisfactorily.
2. I understand that my participation is voluntary and that I am free to withdraw at any time without giving any reason, without my employment being affected.
3. I understand that data collected during the study, may be looked at by individuals from the Universities of Warwick and Birmingham. I give permission for these individuals to have access to my data.
4. I understand that the screening phase of the above study is designed to assess my eligibility for the interventions being offered.
5. I understand that if I’m eligible for an intervention, I will be contacted again by the research team with further information on the specific intervention I may be eligible for and instructions on how to proceed.

**YES NO**

1. Whether eligible or not for the INWORK study, would you like to be contacted by the research team with invitations for future studies? (If yes, proceed to 6.1)
   1. I understand that my name and email address will be stored on the University of Warwick servers for 5 years.

Name of Participant Date Signature

Name of Person Date Signature
