# Supplementary material for: Supporting employers and their employees with Mental hEalth problems to remain eNgaged and producTive at wORk (MENTOR): A feasibility randomised controlled trial protocol
Source: PLoS One. 2023 Apr 20;18(4):e0283598. doi: 10.1371/journal.pone.0283598 (PMC10118171; doi:10.1371/journal.pone.0283598)
Supplement: S3 File — (PDF) [file pone.0283598.s004.pdf]

S1 File.

These are based in part on the Individual Placement Support (IPS) employment specialists and the IPS fidelity scale [17], but with modifications and specifications aimed at supporting people already in employment to maintain and improve their well-being. These have been developed and refined by Mind (the provider organisation) subsequent to internal Mind stakeholder consultation groups. These descriptors are:

1. An ethos that recovery is likely and therefore work can be maintained.

2. Zero exclusion – a person's desire to be employed is the only criterion for access to employment support. Individual characteristics like work history, current mental health symptoms, addiction issues, and convictions do not affect access, but will be used to tailor the intensity of employment support.

3. The support provided is based on employee preferences, within the set boundaries of the role.

4. The MHELW is independent. The worker will provide support in the best interests of the employee. However, the worker will focus on the needs of both the employee and employer - this is based on the idea that it is in the interests of both the employer and employee that the individual be supported to continue to work and be productive.

5. The support to the employee and employer will be individually tailored, but will in general terms involve:

a. Stage 1 - preparatory meetings with both parties where process and desired goals are set; education about mental health, work and productivity will be provided.

b. Stage 2 - involving tailored support regarding advice and support around good workplace wellbeing practices, adaptations to be made and mental health literacy;

c. Stage 3 – review, future planning and MHELW exit plan.

During the trial preparation critical components (e.g., training manual) will be manualised to aid implementation and future replication, but also used as a basis to develop the training programme. The intervention will last for a maximum of 3 months, the point at which primary and secondary outcome measurements will be taken.

We will recruit individuals with a minimum of six months experience of providing mental health and wellbeing services and in providing advice, information and support about workplace wellbeing and positive mental health at work. MHELWs will be trained (jointly with research team members) and employed by the charity Mind (or local Minds).
